# Imperforate Hymen: A Comprehensive Systematic Review

**DOI:** 10.3390/jcm8010056

**Published:** 2019-01-07

**Authors:** Keum Hwa Lee, Ji Sun Hong, Hyuk Jun Jung, Hyun Ki Jeong, Seo Jin Moon, Woo Hyun Park, Yoon Mi Jeong, Seung Won Song, Yongjune Suk, Min Ji Son, Jae Jung Lim, Jae Il Shin

**Affiliations:** 1 Department of Pediatrics, Yonsei University College of Medicine, Yonsei-ro 50, Seodaemun-gu, C.P.O. Box 8044, Seoul 03722, Korea; AZSAGM@yuhs.ac; 2 Division of Pediatric Nephrology, Severance Children’s Hospital, Seoul 03722, Korea; 3 Yonsei University Wonju College of Medicine, Wonju 26426, Korea; g7284@naver.com; 4 Yonsei University College of Medicine, Seoul 03722, Korea; hm96077@yonsei.ac.kr (H.J.J.); sutcost@yonsei.ac.kr (H.K.J.); heypoooo@gmail.com (S.J.M.); dngusng@naver.com (W.H.P.); 29dyd@naver.com (Y.M.J.); seungwon310@gmail.com (S.W.S.); syj94kr@naver.com (Y.S.); minji9144@hanmail.net (M.J.S.); sis02103@naver.com (J.J.L.); 5 Institute of Kidney Disease Research, Yonsei University College of Medicine, Seoul 03722, Korea

**Keywords:** imperforate hymen, abdominal pain, genitourinary symptoms, hymenectomy, hymenotomy, improvement, systematic review

## Abstract

Imperforate hymen (IH) is an uncommon congenital anomaly of the female genital tract, with the hymen completely obstructing the vaginal opening. Despite the simple diagnosis and treatment of IH, missed or delayed diagnosis is often a clinical problem owing to its low incidence, nonspecific symptoms, or insufficient physical examination. The aim of this study is to identify the characteristics, clinical presentations, treatment modalities, and outcomes of imperforate hymen patients. In this study, a literature search of PubMed databases was performed for sources published up to 3 July 2018 for English-language studies with the term “imperforate hymen”. The literature review identified 251 citations and 155 articles (143 case reports, 12 case series) containing 253 patients who were finally included (two papers were not written in English). Among 236 postnatal patients, the mean age of the patients was 10.7 ± 4.7 years. Abdominal pain (54.2%), urinary retention (20.3%), abnormal menstruation (14.0%), dysuria (9.7%), increased urinary frequency (5.1%), severe presentation of renal failure (*n* = 5, 2.1%), and urinary tract infection (*n* = 1, 0.4%) were presented. Most patients diagnosed with the condition underwent surgical treatment (83.5%), most of whom were treated via a hymenotomy (35.2%) and hymenectomy (36.4%), and the use of prophylactic antibiotics were only used in 7 patients. There were no differences in outcomes between two surgical methods. In addition, 141 (59.7%) patients showed improvement and 5 deceased patients were not related to IH or the operation itself; Complications, such as vaginal adhesion, were only noted in 6.6% of patients. In addition, among 17 cases of newborns with a diagnosis of IH before birth, hymenectomy (*n* = 5, 29.4%) and hymenotomy (*n* = 9, 52.9%) were the main treatment modalities and showed improved prognosis in 52.9% of newborns. Because IH diagnosis is easy and postsurgical prognosis is good, clinicians should carefully examine every female patient at birth. IH should be considered regarding adolescent girls with abdominal pain, lower back pain, or urinary retention, and perform appropriate physical examinations of the genital introitus. In addition, accurate diagnosis as IH, not misdiagnosing as vaginal septum or agenesis, is important to prevent severe complications such as stricture and ascending infection.

## 1. Introduction 

Imperforate hymen (IH) is an uncommon congenital anomaly of the female genital tract, in which the hymen completely obstructs the vaginal opening, with an approximate incidence of 0.05–0.1% [1,2]. IH obstructs uterine and vaginal secretions (also called hematocolpos), causing amenorrhea and cyclic pelvic pain [2]. IH may be associated with other developmental anomalies [3], but some reports propose that it is not generally related to Müllerian anomalies, and evaluating urogenital anomalies is unnecessary [4]. There have been rare cases of familial IH occurrence; most cases are thought to occur sporadically and no genetic mutations have been identified [5]. 

IH is often diagnosed in adolescent girls after menarche, mainly presenting with amenorrhea and lower abdominal pain or urinary retention [6]. Most young girls with IH are asymptomatic and diagnosed incidentally until menarche. Rarely, however, especially in newborns, the fetus’ secretion by maternal estrogen may cause hydrocolpos and hydrometrocolpos, presenting as abdominal mass in 0.006% of female neonates [7]. 

IH can be diagnosed by inspecting the external genitalia, which presents a bulging, bluish hymenal membrane [1], but an abdominal ultrasound may accurately show a pelvic cystic mass [1]. Although IH is a benign congenital disorder, late detection and diagnosis may result in severe morbidity and requirement of additional interventions [2]. Without proper management, IH can cause infections, subfertility, endometriosis, or hydronephrosis and renal failure in rare cases [8]. The treatment of choice is based on cruciate incision or excision of the hymen [1]. In contrast to hymenectomy with X, T, cross, or crucial incision, and resection of excess tissues, hymen-preserving surgeries, such as a simple vertical incision and annular hymenotomy, can be an option for patients desiring virginity [9,10]. Alternative treatments include a carbon dioxide laser or insertion of a Foley catheter [11,12]. 

Despite the simple diagnosis and treatment of IH, missed/delayed diagnosis is a critical issue owing to low incidence, nonspecific symptoms, or insufficient physical examination. Therefore, when adolescent girls present with abdominal pain or acute urinary retention, clinicians must suspect IH and conduct thorough abdominal and gynecological examinations. 

Although there have been many case reviews and reports, no systematic review on IH has been reported. Therefore, we searched 253 cases of IH and reviewed the epidemiology, symptoms, treatment options, and clinical outcomes. We also aimed to raise awareness among clinicians by discussing several cases of IH with severe complications. 

## 2. Methods

### 2.1. Search Strategy

**Figure 1:**
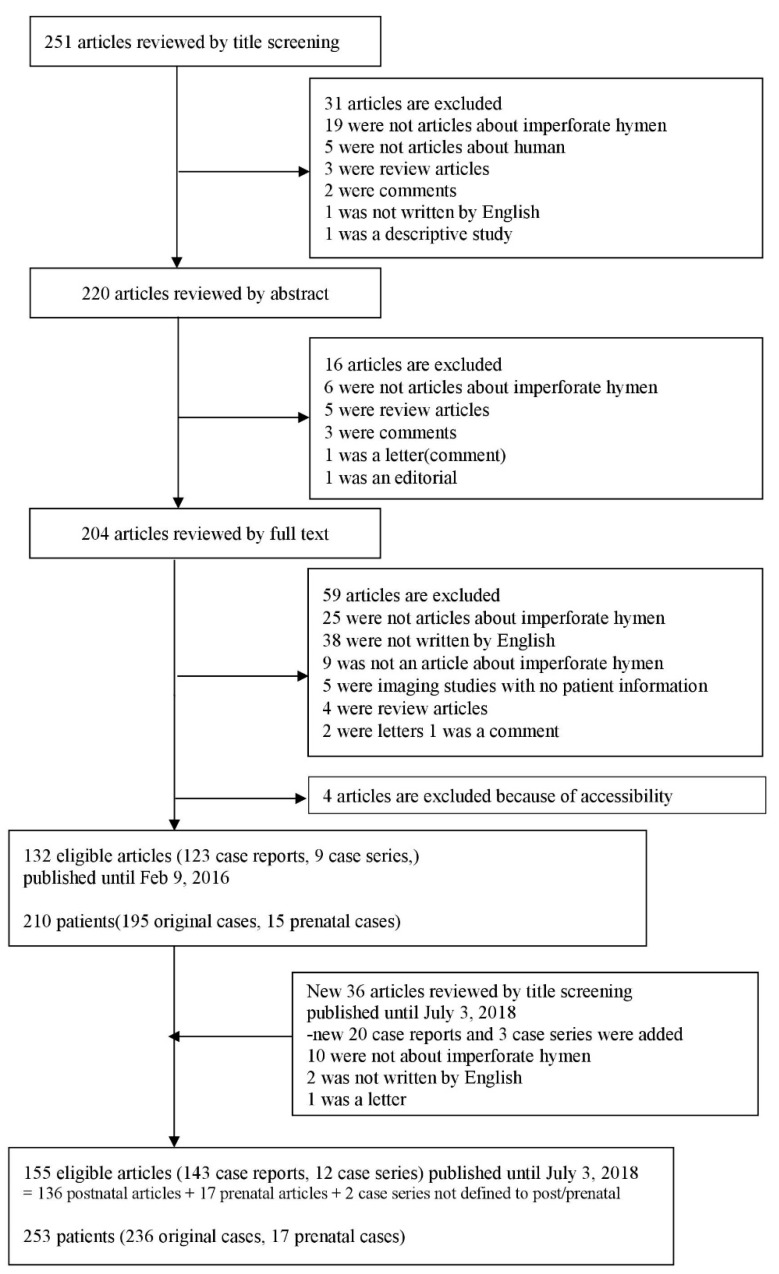
Flow chart of literature search.

We searched PubMed for sources published up until 3 July 2018 using the keyword “imperforate hymen”. We included studies if they met the following criteria: (I) published in a peer-reviewed journal with accessible full-length content in English, (II) included patients with IH, (III) presented symptoms or imaging findings, (IV) evaluated treatment, and (V) reported on relevant outcomes. The identification of literature was conducted in accordance with the PRISMA (Preferred Reporting Items for Systematic Reviews and Meta-analyses) Statement [13] (Supplementary Table S1). Finally, 155 eligible articles (143 case reports, 12 case series) were identified for the systematic review of IH. We reorganized the eligible articles into two groups: (1) articles with postnatal patients (136 articles, 236 cases, Supplementary Table S2); and (2) articles with prenatal diagnosis (17 articles, 17 cases, Supplementary Table S3). Two case series are not belonged to either postnatal or prenatal groups, not containing detailed patient information (Supplementary Table S4). Articles in each group were reviewed and analyzed entirely. Details of the selection process are presented in Figure 1.

### 2.2. Extraction of Data

We have presented the data extracted from each case report or case series in Supplementary Tables S2 and S3. For each article, we extracted information on the first author’s surname, journal name, year of publication, country, study design, age of patients (mean age of patients in case series), patient’s country, gynecological history, presented symptoms and period thereof, combined abnormality, number of doctor(s) for diagnosis, treatment, amount of drained fluid after treatment (mL), and outcomes. In articles that reported prenatally diagnosed patients, we recorded the information concerning the mother’s age and parity, gestational age of the patients at diagnosis, presented problems, used imaging modality and findings, and combined abnormality especially in the urinary system. Discrepancies were resolved through a discussion.

## 3. Results 

### 3.1. Characteristics of Patients

The 136 eligible articles with postnatal diagnosis were selected according to the inclusion/exclusion criteria. A total of 236 patients classified according to various variables have been listed in Table 1. Variables such as age, sex, regional distribution, perinatal diagnosis, the number of doctors for diagnosis, gynecological history, and combined abnormality were studied. The mean age of the patients was 10.7 ± 4.7 years. The majority of the patients were in their adolescence (*n* = 153, 64.8%), and 33 of them were in their childhood (14.0%). Only 11 patients (4.7%) were adults (older than 18 years). Most of the cases were reported in America (*n* = 105, 44.5%), followed by Europe (*n* = 52, 22.0%). Only 26 patients (11.0%) were diagnosed in their perinatal period, and in the rest of them, early diagnosis was missed. Further, 22 patients (9.3%) had a familial history of IH. We found that five patients came to the hospital because of child abuse and they were first diagnosed during the physical examination process. One of them was sexually abused but her hymen was intact in keeping with the imperforation status. In one case, the patient was misdiagnosed to have Müllerian agenesis and was treated using a vaginal dilator. In 48 cases, other anomalies, such as anorectal/vaginal atresia, urethrovaginal fistula, cloaca, absent radius, and McKusick–Kaufman syndrome, accompanied IH. More information about the patients is presented in Table 1.

### 3.2. Clinical Presentation

Clinical presentation of the patients with IH is shown in Table 2. The most frequent symptom of the patients was abdominal pain (*n* = 128, 54.2%). Further, a large number of patients experienced genitourinary symptoms, including urinary retention (*n* = 48, 20.3%), abnormal menstruation (*n* = 33, 14.0%), dysuria (*n* = 23, 9.7%), and urinary frequency (*n* = 12, 5.1%). Six patients showed severe presentation of renal failure (*n* = 5, 2.1%) and urinary tract infection (*n* = 1, 0.4%). In some cases, patients presented symptoms that could not easily be associated with IH, e.g., a palpable mass (*n* = 23, 9.7%), back pain (*n* = 21, 8.9%), and respiratory distress (*n* = 2, 0.8%). 

### 3.3. Treatment

A majority of the patients received surgical therapy (*n* = 197, 83.5%), most of whom were treated with a hymenotomy (*n* = 83, 35.2%) or hymenectomy (*n* = 86, 36.4%). In some cases, patients also needed to receive additional surgeries such as vaginal septum repair, vaginoplasty, or closure of fistula. Nine patients received medical therapies. Eight of them were administered prophylactic antibiotics or treated via irrigation of the vaginal cavity with an antibiotic solution in combination with surgical therapies. A GnRH agonist was used as a mono-therapeutic agent in an 18-month-old Asian patient who had a history of central precocious puberty combined with vaginal atresia. Two patients were found to have observational follow-ups without interventions. Three patients received no treatment at all, and were spontaneously cured. In those undergoing surgery, this was the only mode of treatment for 68.2% cases (*n* = 161); however, some treatments that are mentioned in Table 3 were used in combination. In all, 8.1% patients received a combination of two therapies, e.g., hymenectomy and vaginal septum repair (*n* = 3, 1.3%) and hymenotomy and prophylactic antibiotics (*n* = 4, 1.6%). In three patients, three kinds of therapies were used in combination (1.3%). Detailed information is shown in Supplementary Table S5.

### 3.4. Outcomes and Characteristics

We could not obtain information about outcomes in 31.8% patients. Of the remaining, improvement was seen in 141 patients (59.7%), whereas 15 patients (6.6%) had complications and 5 (2.1%) died (Table 4).

Outcomes of patients have been described in Table 5. Among those with improvement (*n* = 141), only 9.2% were diagnosed at the perinatal stage, with 71.6% diagnosed in their adolescence (12–18 years). Most patients had no combined abnormality (*n* = 116, 82.3%) such as bilateral hydronephrosis, transverse vaginal septum, and bicornuate uterus. Abdominal pain (*n* = 86, 61.0%) and urinary retention (*n* = 39, 27.7%) were the most common clinical symptoms, while there were no cases of urinary tract infections. Hymenectomy (*n* = 66, 46.8%) and hymenotomy (*n* = 66, 46.8%) were the main treatment modalities for IH. 

There were 15 patients with complications including vaginal adhesion. These patients had characteristics similar to those of patients with improvement. In line with those having an improvement, hymenectomy (*n* = 9, 60.0%) and hymenotomy (*n* = 3, 20.0%) were the main treatment forms. Hymenectomy, however, showed more frequent complications than hymenotomy. Abdominal pain (*n* = 11, 73.3%) was still the most common symptom with no bladder distension or urinary tract infections, but amenorrhea (*n* = 4, 26.7%) and dysuria (*n* = 3, 20.0%) were common symptoms. 

Patients who died during follow-up (*n* = 5) showed different characteristics compared with the abovementioned patients. They were all diagnosed at their perinatal stage and combined abnormalities were observed. This group of patients could not survive until their infant stage (1 month to 2 years) mainly owing to cardiorespiratory distress (*n* = 3).

### 3.5. Imperforate Hymen before Birth

We included 17 cases of newborns with a diagnosis of IH before birth (Table 6). The largest proportion of these patients were preterm newborns (<38 weeks) (*n* = 13, 76.5%), followed by normal newborns (38–42 weeks) (*n* = 3, 17.6%). However, there was no post-term newborn (>42 weeks) with IH. Abnormalities were diagnosed before delivery in 16 newborns. Most of them had a single abnormality (*n* = 9, 52.9%), which was hydrometrocolpos (*n* = 5, 29.4%) in most cases. Hymenectomy (*n* = 5, 29.4%) and hymenotomy (*n* = 9, 52.9%) were the main treatment modalities and showed improved prognosis in 52.9% of newborns. However, one patient (*n* = 1, 5.9%) showed a complication of asymmetric renal function and residual functional dilation and one (5.9%) died of urosepsis.

## 4. Discussion

The hymen is the junction of the urogenital sinus and the sinovaginal bulbs. In embryonic stages, the hymen is perforated to make a connection between the vestibule and the vaginal canal. If this stage fails, individuals are born with IH [15,16]. IH is a rare disease, with an estimated incidence rate of 0.05%–0.1% [17]. Generally, this rare congenital anomaly is diagnosed during adolescence after menarche, presenting with abdominal pain and amenorrhea. IH does not present itself as an abdominal mass during the perinatal period [15], and most patients with IH are asymptomatic and not diagnosed until menarche. Our study also showed that among 253 patients included in the reviewed articles, 236 patients were diagnosed after birth and only 17 (6.7%) were diagnosed during their prenatal period. Early diagnosis was missed in the remaining patients. Most patients presented with abdominal pain (54.2%) and many patients had urinary retention (20.3%), abnormal menstruation (*n* = 33, 14%), dysuria (9.7%), and urinary frequency (*n* = 12, 5.1%). 

Delayed diagnosis is mainly associated with the asymptomatic period for a long time as it is painless during the accumulation of menstrual blood. IH is a rare disease that may not be detected until the onset of menses, when the accumulation of menstrual blood in the uterus and vagina (called hematocolpos) develops symptoms resulting from its mechanical effect on the bladder and urethra [18,19,20]. If left untreated, this condition can also cause obstructive urinary symptoms, constipation, or dysuria [9]. However, IH can be detected for diagnosis in adolescent girls with abdominal pain, even before menarche. 

In our study, 26 (11.0%) patients were newborns and 72 (30.5%) were under 12 years of age. This indicates that IH can be readily diagnosed by inspecting the external genitalia. If uncertainties remain, ultrasonography or magnetic resonance imaging can help ease the diagnosis [1]. However, clinicians rarely conduct appropriate physical examinations and take a detailed gynecological history because of the low incidence and nonspecific symptoms of IH. Therefore, diagnosis of IH is missed or delayed in most cases [6]. 

Choice of treatment is based on hymenectomy (cruciate incision or excision of hymen) [8]. Hymen-preserving surgeries, such as simple vertical incision and annular hymenotomy, can be an option for patients desiring virginity [9,10]. Alternative treatments include carbon dioxide laser or insertion of a Foley catheter [10,12]. In our study, most patients received surgical therapy (83.5%), most of whom were treated with a hymenotomy (35.2%) or hymenectomy (36.4%). Surgery was the main single treatment option (68.2%); however, 8.1% patients received two kinds of therapies, e.g., a combination of hymenectomy and vaginal septum repair (1.3%). Three patients received three kinds of therapies (1.3%). In cases with a conservative approach, no improvement was observed. There was no difference of improved outcome between hymenotomy and hymenectomy, and use of prophylactic antibiotics were only identified in six patients. Besides, complications such as reclosure, vaginal adenosis, or vaginal adhesion were only noted in 6.6% patients. According to our data (Table 5) and previous several papers, hymenotomy can be enough for reducing these complications [9,18,21]. Thus, we propose that early diagnosis, detection of IH, and minimal preserving hymen surgery are of importance. Although IH is a benign congenital disorder, it can cause endometriosis, subfertility, infection, or hydronephrosis and renal failure without proper management.

Among patients described in case reports, there were five patients with transverse vaginal septum combined with imperforate hymen [22,23,24,25,26] and one patient with uterocervicovaginal septum [27]. Also, there was one patient who was a neonate at the time of diagnosis with sacral agenesis [3] not fully separated with an imperforate hymen. However, no case reports described misdiagnosis other diseases as an imperforate hymen. Other gynecological diseases, especially vaginal septum and agenesis, require much more complex care and should not be confused with IH, which is fairly easy to correct and not associated with as many or severe complications such as stricture and ascending infection. Clinicians should always keep in mind both of the possibilities that the IH may have been combined with these diseases and that other diseases have been misidentified as IH.

There are several potential limitations in this review. It can be possible that several studies were not detected although we comprehensively selected researches across multiple databases. In addition, we could only include data on information provided in case reports and series. Due to the characteristics of a systematic review, this article represents a synthesis of the opinions of the authors who wrote the case reports or case series, rather than new concepts or information about this disease. 

In conclusion, IH is a rare disease for which early diagnosis is easy to miss. IH can cause acute urinary retention. However, the diagnosis is easy and postsurgical prognosis is good. Therefore, clinicians, especially urologists, gynecologists, or pediatricians, should carefully examine every female patient at birth. Even when the detection is delayed until adolescence, IH should be considered a possibility in patients who complain of abdominal pain, lower back pain, or urinary retention, and assessed by conducting prompt and appropriate physical examinations of the genital introitus. Moreover, it is important to diagnose IH accurately by not misdiagnosing it as vaginal septum or agenesis to prevent severe complications of wrong treatment. Further prospective and bigger studies would be necessary addressing the issue of the specificity of the outcome according to the each treatment in the future.

## Figures and Tables

**Table 1:** Characteristics of case-reported patients with imperforate hymen.

Variables	Total Number of Patients (*n* = 236)
	Number of Patients (%)
**Age**	
Neonate (<1 month)	26 (11.0%)
Infant (1 month–2 years)	13 (5.5%)
Child (2 years–12 years)	33 (14.0%)
Adolescent (12 years–18 years)	153 (64.8%)
Adult (>18 years)	11 (4.7%)
**Sex**	
Female	236 (100%)
Male	0 (0.0%)
**Patient’s country**	
Asia	37 (15.7%)
Europe	52 (22.0%)
Africa	13 (5.5%)
America	105 (44.5%)
Middle east	29 (12.3%)
**The number of doctors for diagnosis**	
One	94 (40.0%)
More than one *	45 (19.1%)
**Gynecological history**	
Familial history of imperforate hymen	22 (9.3%)
Menstruation	8 (3.4%)
Hymenotomy or hymenectomy	6 (2.5%)
Abuse	5 (2.1%)
Central precocious puberty	2 (0.8%)
Previously hymen opened	1 (0.4%)
Labial adhesion	1 (0.4%)
Misdiagnosed as Müllerian agenesis treated with vaginal dilator	1 (0.4%)
**Multiple abnormality** †	
Yes	48 (20.3%)
No	188 (79.7%)

* More than one doctor refers to 2 (*n* = 34), 3 (*n* = 8), 4 (*n* = 1), 6 (*n* = 1), or more than 7 doctors (*n* = 1). † Multiple abnormalities include absent left radius, angiomyolipoma arises primarily in the kidneys, anorectal atresia, bicornuate uterus, bilateral duplex ureter, bilateral hydronephrosis, cloaca, congenital heart defects, didelphic uterus with or without double vagina, double genital system, duplex kidney, Escobar syndrome, hematocolpometra, high aortic bifurcation, imperforate anus, ipsilateral renal agenesis, labial adhesion, Langer–Giedion syndrome, left hypoplastic kidney with ectopic ureter, McKusick–Kaufman syndrome (MKKS), mild subluxation of the hips, persistent cloaca, primary vaginal calculus, right absent ureter, right cystic dysplastic kidney, rupture of bladder, sacral agenesis, systemic aplasia cutis congenital, transverse vaginal septum, ulnar mammary syndrome, unicornuate uterus, urethrovaginal fistula, vaginal atresia, and vaginal occlusion by an imperforate hymen.

**Table 2:** Clinical presentation of case-reported patients with imperforate hymen.

Clinical Presentation	Total Number of Patients (*n* = 236)
	Number of Patients (%)
**General condition**	
Fever	5 (2.1%)
Hot flushes	2 (0.8%)
Lethargy	3 (1.3%)
Loss of appetite	2 (0.8%)
General discomfort	2 (0.8%)
Weight gain	1 (0.4%)
Poor feeding	1 (0.4%)
**Cardiovascular**	
Sudden chest pain with swelling of the upper torso	1 (0.4%)
Tachypnea	1 (0.4%)
**Palpable mass**	23 (9.7%)
**Respiratory**	
Respiratory distress	2 (0.8%)
Rapid breathing	1 (0.4%)
Thoracoabdominal respiration	1 (0.4%)
**Gastrointestinal**	
Abdominal pain	128 (54.2%)
Abdominal discomfort	10 (4.2%)
Abdominal distension	18 (7.6%)
Constipation	13 (5.5%)
Diarrhea	3 (1.3%)
Vomiting	5 (2.1%)
Nausea	5 (2.1%)
Tenesmus	2 (0.8%)
Fecal frequency and urgency	1 (0.4%)
Inability to pass meconium	1 (0.4%)
Peritoneal sign	1 (0.4%)
**Genitourinary**	
Urinary retention	48 (20.3%)
Urinary frequency	12 (5.1%)
Urinary incontinence	4 (1.7%)
Urinary urgency	2 (0.8%)
Urinary hesitancy	1 (0.4%)
Nocturia	2 (0.8%)
Renal failure	5 (2.1%)
Urinary tract infection	1 (0.4%)
Bladder distension	1 (0.4%)
Straining during urination	1 (0.4%)
Polyuria	1 (0.4%)
Oliguria	1 (0.4%)
Dysuria	23 (9.7%)
Abnormal menstruation *	33 (14.0%)
Heavy vaginal bleeding	1 (0.4%)
Others	10 (4.2%)
Perineal pain and pruritus	1 (0.4%)
Perineal bulge	7 (3.0%)
Large interlabial swelling	1 (0.4%)
Vaginal irritation/tenderness/fullness/swelling	4 (1.6%)
Problems in sexual intercourse	1 (0.4%)
**Musculoskeletal**	
Back pain	21 (8.9%)
Leg pain	2 (0.8%)
Lower limb swelling	2 (0.8%)
**Neurologic**	
Headache	3 (1.2%)
Epileptic attacks	1 (0.4%)
Syncope	1 (0.4%)
**Psychiatric**	
Irritability	1 (0.4%)
**Others** †	15 (6.4%)

* Abnormal menstruation refers to unknown abnormal menstruation (*n* = 1), scanty menstruation (*n* = 1), dysmenorrhea (*n* = 1), and amenorrhea (*n* = 30). † Others refer to primary infertility (*n* = 1), dyspareunia (*n* = 2), nonimmune hydrops fetalis (*n* = 1), anorectal atresia (*n* = 1), multiple anomalies (*n* = 3), appendicitis (*n* = 3), pelvic pressure (*n* = 1), suprapubic pain (*n* = 1), appearance of pubic hair and breast enlargement (*n* = 1), and breast tenderness (*n* = 1).

**Table 3:** Treatment of case-reported patients with imperforate hymen.

Treatment	Total Number of Patients (*n* = 236)
	Number of Patients (%)
**Surgical therapy**	**197 (83.5%)**
Hymenotomy	83 (35.2%)
Hymenectomy	86 (36.4%)
Laparotomy	5 (2.1%)
Vaginal septum repair	4 (1.7%)
Vaginoplasty	1 (0.4%)
Vaginal canal extraction	1 (0.4%)
Vaginal septoplasty	1 (0.4%)
Vaginal orifice dilatation	3 (1.3%)
Perforation and dilatation of hymen	1 (0.4%)
Puncture of hymen	1 (0.4%)
Unknown surgical correction	1 (0.4%)
Surgical incision of the imperforate hymen	1 (0.4%)
Excision of fistula	1 (0.4%)
Simple closure of fistula	1 (0.4%)
Repairing urethrovaginal fistula	1 (0.4%)
Removal of calculi in pelvis	1 (0.4%)
Laparoscopic adhesiolysis	2 (0.8%)
Bladder suturing	1 (0.4%)
Anal cut back operation	1 (0.4%)
Abdominoperineal pull-through	1 (0.4%)
**Medical therapy** *	**9 (3.8%)**
Prophylactic antibiotics	7 (3.0%)
Irrigation of vaginal cavity with antibiotic solution	1 (0.4%)
GnRH agonist	1 (0.4%)
**Observation**	**2 (0.8%)**
**None (self-limited)**	**3 (1.2%)**
**Unknown**	**1 (0.4%)**

* Except GnRH agonist, all the medical therapies were used in combination with surgical therapies. A GnRH agonist was used as a mono-therapeutic agent in an 18-month-old Asian patient [14]. The patient had a history of central precocious puberty and a combined abnormality of vaginal atresia. The patient’s chief complaint was the appearance of pubic hair and breast enlargement.

**Table 4:** Outcomes of case-reported patients with imperforate hymen.

Outcome	Total Number of Patients (*n* = 236)
	Number of Patients (%)
Improved	141 (59.7%)
Complicated *	15 (6.6%)
Died †	5 (2.1%)
Unknown	75 (31.8%)

* Complication includes reclosure (*n* = 4), vaginal adenosis (*n* = 2), vaginal adhesion (*n* = 1), destruction of the urethral sphincter and bladder (*n* = 1), development of cicatricial stenosis in upper vagina (*n* = 1), vaginal canal adhesion (*n* = 1), amenorrhea (*n* = 1), second hymenotomy (*n* = 1), fusion of vaginal septum (*n* = 1), and unknown (*n* = 2). Strictly, amenorrhea (*n* = 1) is not a complication of the operation but we inserted this patient into the complicated cases because the patient showed no menstruation for 1 year after surgery in one article. † Cause of death was cardiorespiratory distress (*n* = 3), sepsis and acute kidney injury (*n* = 1), or asphyxia and dehydration (*n* = 1).

**Table 5:** Outcomes of case-reported patients with imperforate hymen.

Variables	Total Number of Improved Patients (*n* = 141)	Total Number of Complicated * Patients (*n* = 15)	Total Number of Deceased † Patients (*n* = 5)
	Number of Patients (%)	Number of Patients (%)	Number of Patients (%)
**Age**			
Neonate, prenatal (<1 month)	13 (9.2%)	1 (6.7%)	5 (100.0%)
Infant (1 month–2 years)	5 (3.5%)	1 (6.7%)	0 (0.0%)
Child (2 years–12 years)	13 (9.2%)	2 (13.3%)	0 (0.0%)
Adolescent (12 years–18 years)	101 (71.6%)	9 (60.0%)	0 (0.0%)
Adult (>18 years)	9 (6.4%)	2 (13.3%)	0 (0.0%)
**Patient’s country**			
Asia	27 (19.1%)	3 (20.0%)	1 (20.0%)
Europe	28 (19.9%)	4 (26.7%)	3 (60.0%)
Africa	8 (5.7%)	1 (6.7%)	1 (20.0%)
America	54 (38.3%)	7 (46.7%)	0 (0.0%)
Middle east	24 (17.0%)	0 (0.0%)	0 (0.0%)
**The number of doctors for diagnosis** ‡			
One	67 (47.5%)	9 (60.0%)	-(-%)
More than one	37 (26.2%)	3 (20.0%)	-(-%)
**Gynecological history**			
Familial history of imperforate hymen	14 (9.9%)	0 (0.0%)	0 (0.0%)
Menstruation	5 (3.5%)	1 (6.7%)	0 (0.0%)
Hymenotomy or hymenectomy	5 (3.5%)	0 (0.0%)	0 (0.0%)
Abuse	0 (0.0%)	0 (0.0%)	0 (0.0%)
Central precocious puberty	2 (1.4%)	0 (0.0%)	0 (0.0%)
Labial adhesion	1 (0.7%)	0 (0.0%)	0 (0.0%)
**Combined abnormality** **			
Yes	26 (18.4%)	3 (20.0%)	5 (100.0%)
Transverse vaginal septum	4 (2.8%)	1 (6.7%)	0 (0.0%)
No	116 (82.3%)	12 (80.0%)	0 (0.0%)
**Clinical presentations**			
Amenorrhea	21 (14.9%)	4 (26.7%)	0 (0.0%)
Abdominal pain	86 (61.0%)	11 (73.3%)	0 (0.0%)
Abdominal discomfort	5 (3.5%)	1 (6.7%)	0 (0.0%)
Back pain	13 (9.2%)	1 (6.7%)	0 (0.0%)
Abdominal distension	13 (9.2%)	1 (6.7%)	1 (20.0%)
Bladder distension	3 (2.1%)	0 (0.0%)	0 (0.0%)
Urinary frequency	10 (7.1%)	0 (0.0%)	0 (0.0%)
Urinary retention	39 (27.7%)	1 (6.7%)	1 (20.0%)
Constipation	4 (2.8%)	1 (6.7%)	0 (0.0%)
Headache	2 (1.4%)	1 (6.7%)	0 (0.0%)
Dysuria	11 (7.8%)	3 (20.0%)	0 (0.0%)
Renal failure	5 (3.5%)	0 (0.0%)	0 (0.0%)
Urinary tract infection (UTI)	0 (0.0%)	0 (0.0%)	0 (0.0%)
Palpable mass	12 (8.5%)	2 (13.3%)	0 (0.0%)
**Treatments**			
Hymenectomy	66 (46.8%)	9 (60.0%)	1 (20.0%)
Hymenotomy	66 (46.8%)	3 (20.0%)	0 (0.0%)
Vaginoplasty	1 (0.7%)	0 (0.0%)	0 (0.0%)
Vaginal septum repair	4 (2.8%)	2 (13.3%)	0 (0.0%)
Vaginal orifice dilatation	2 (1.4%)	1 (6.7%)	0 (0.0%)
Observation	0 (0.0%)	0 (0.0%)	0 (0.0%)
Prophylactic antibiotics	5 (3.5%)	1 (6.7%)	0 (0.0%)
Laparotomy	4 (2.8%)	0 (0.0%)	0 (0.0%)

* Complication includes reclosure (*n* = 4), vaginal adenosis (*n* = 2), vaginal adhesion (*n* = 1), destruction of the urethral sphincter and bladder (*n* = 1), development of cicatricial stenosis in upper vagina (*n* = 1), vaginal canal adhesion (*n* = 1), amenorrhea (*n* = 1), second hymenotomy (*n* = 1), fusion of vaginal septum (*n* = 1), and unknown (*n* = 2). Strictly, amenorrhea (*n* = 1) is not a complication of the operation but we inserted this patient into the complicated cases because the patient showed no menstruation for 1 year after surgery in one article. † Causes: Cardiorespiratory distress (*n* = 3), Sepsis and acute kidney injury (*n* = 1) and Asphyxia and dehydration (*n* = 1). ‡ There is no explanation of the number of doctors in deceased patients. ** Combined abnormality contains bilateral hydronephrosis, transverse vaginal septum, bicornuate uterus, etc.

**Table 6:** Demographics of case-reported newborns diagnosed with imperforate hymen before birth.

Variables	Total Number of Patients (*n* = 17)
	Number of Patients (%)
**Maternal age (years)**	
<20	1 (5.9%)
20–24	0 (0.0%)
25–29	5 (29.4%)
30–35	3 (17.6%)
>35	6 (35.3%)
Unknown	2 (11.8%)
**Gravidity**	
Primipara	7 (41.2%)
Multipara	7 (41.2%)
Unknown	3 (17.6%)
**Gestational age (wks)**	
Preterm (<38)	13 (76.5%)
Normal (38–42)	3 (17.6%)
Post-term (>42)	0 (0.0%)
Unknown	1 (5.9%)
**Diagnosed abnormality** * **before born**	
**Nothing diagnosed** †	**1 (5.9%)**
**Single kinds of abnormalities**	**9 (52.9%)**
Hydrocolpos	1 (5.9%)
Hydrometrocolpos	5 (29.4%)
Fetal pelvic cyst/mass	2 (11.8%)
Hydronephrosis	1 (5.9%)
**Two kinds of abnormalities**	**5 (29.4%)**
Hydrocolpos + Fetal pelvic cyst/mass	1 (5.9%)
Hydrometrocolpos + Fetal pelvic cyst/mass	1 (5.9%)
Fetal pelvic cyst/mass + Hydronephrosis	2 (11.8%)
Fetal pelvic cyst/mass + Kidney pelvic dilatation	1 (5.9%)
**Three kinds of abnormalities**	**2 (11.8%)**
Hydrocolpos + Fetal pelvic cyst/mass + Hydronephrosis	2 (11.8%)
**Treatment**	
Hymenectomy	5 (29.4%)
Hymenotomy	9 (52.9%)
Others ‡	3 (17.6%)
**Outcome**	
Improved	9 (52.9%)
Complicated	1 (5.9%)
Died	1 (5.9%)
Unknown	6 (35.3%)

* Hydrocolpos, hydrometrocolpos, fetal pelvic cyst/mass, and hydronephrosis. † Fetal ascites and distended uterus and vagina was diagnosed by ultrasonography finding. ‡ One patient self-limited, another patient did only observation, and the other patient did not know what treatment they underwent.

## Supplementary Materials

The following are available online at http://www.mdpi.com/2077-0383/8/1/56/s1, Table S1: Checklist summarizing compliance with PRISMA guidelines, Table S2: List of case-reported characteristics including presenting symptoms as well as treatment outcomes and comorbidities, Table S3: List of perinatal case-reported characteristics including presenting symptoms as well as treatment outcomes and comorbidities, Table S4: Summary profiles of case-series reports about IH (not describe detail information of patients), Table S5: Combination of treatments for case-reported patients with imperforate hymen.
