# Supplementary material for: Imperforate Hymen: A Comprehensive Systematic Review
Source: J Clin Med. 2019 Jan 7;8(1):56. doi: 10.3390/jcm8010056 (PMC6352236; doi:10.3390/jcm8010056)
Supplement: Supplementary file 1 [file jcm-08-00056-s001.pdf]

Supplementary Tables

Imperforate Hymen: A Comprehensive Systematic Review

Keum Hwa Lee[1,2*], Ji Sun Hong[3*], Hyuk Jun Jung[4], Hyun Ki Jeong[4], Seo Jin Moon[4], Woo Hyun Park[4], Yoon Mi Jeong[4], Seung Won Song[4], Yongjune Suk[4], Min Ji Son[4], Jae Jung Lim[4], and Jae Il Shin[1,2,5]

[1]Department of Pediatrics, Yonsei University College of Medicine, Yonsei-ro 50, Seodaemun-gu, C.P.O. Box 8044, Seoul 03722, Republic of Korea; AZSAGM@yuhs.ac (K.H.L.)

[2]Department of Pediatric Nephrology, Severance Children's Hospital, Seoul 03722, Republic of Korea

[3]Yonsei University Wonju College of Medicine, Wonju 26426, Republic of Korea; g7284@naver.com (J.S.H)

[4]Yonsei University College of Medicine, Seoul 03722, Republic of Korea; hm96077@yonsei.ac.kr (H.J.J); sutcost@yonsei.ac.kr (H.K.J); heypoooo@gmail.com (S.J.M); dngusng@naver.com (W.H.P); 29dyd@naver.com (Y.M.J); seungwon310@gmail.com (S.W.S); syj94kr@naver.com (S.Y.J); minji9144@hanmail.net (M.J.S); sis02103@naver.com (J.J.L)

[5]Institute of Kidney Disease Research, Yonsei University College of Medicine, Seoul 03722, Republic of Korea

Corresponding Author: Jae Il Shin, M.D., Ph.D.

Address: 50 Yonsei-ro, Seodaemun-gu, C.P.O. Box 8044, Department of Pediatrics, Yonsei University College of Medicine, Seoul 120-752, Republic of Korea. E-mail:shinji@yuhs.ac; Tel.:+82-2-2228-2050; Fax: +82-2-393-9118

Supplementary Table S1. Checklist summarizing compliance with PRISMA guidelines

Topic	#	Checklist item	Reported on page #
**TITLE**			
Title	1	Identify the report as a systematic review, meta-analysis, or both.	1
**ABSTRACT**			
Structured summary	2	Provide a structured summary including, as applicable: background; objectives; data sources; study eligibility criteria, participants, and interventions; study appraisal and synthesis methods; results; limitations; conclusions and implications of key findings; systematic review registration number.	2
**INTRODUCTION**			
Rationale	3	Describe the rationale for the review in the context of what is already known.	3
Objectives	4	Provide an explicit statement of questions being addressed with reference to participants, interventions, comparisons, outcomes, and study design (PICOS).	3-4
**METHODS**			
Protocol and registration	5	Indicate if a review protocol exists, if and where it can be accessed (e.g., Web address), and, if available, provide registration information including registration number.	N/A
Eligibility criteria	6	Specify study characteristics (e.g., PICOS, length of follow-up) and report characteristics (e.g., years considered, language, publication status) used as criteria for eligibility, giving rationale.	4-5
Information sources	7	Describe all information sources (e.g., databases with dates of coverage, contact with study authors to identify additional studies) in the search and date last searched.	4-5
Search	8	Present full electronic search strategy for at least one database, including any limits used, such that it could be repeated.	4-5
Study selection	9	State the process for selecting studies (i.e., screening, eligibility, included in systematic review, and, if applicable, included in the meta-analysis).	4-5 (Figure1)
Data collection process	10	Describe method of data extraction from reports (e.g., piloted forms, independently, in duplicate) and any processes for obtaining and confirming data from investigators.	4-5
Data items	11	List and define all variables for which data were sought (e.g., PICOS, funding sources) and any assumptions and simplifications made.	Supplementary Tables and Tables
Risk of bias in individual studies	12	Describe methods used for assessing risk of bias of individual studies (including specification of whether this was done at the study or outcome level), and how this information is to be used in any data synthesis.	N/A
Summary measures	13	State the principal summary measures (e.g., risk ratio, difference in means).	N/A
Synthesis of results	14	Describe the methods of handling data and combining results of studies, if done, including measures of consistency (e.g., I^2) for each meta-analysis.	N/A

Section/topic	#	Checklist item	Reported on page #
Risk of bias across studies	15	Specify any assessment of risk of bias that may affect the cumulative evidence (e.g., publication bias, selective reporting within studies).	N/A
Additional analyses	16	Describe methods of additional analyses (e.g., sensitivity or subgroup analyses, meta-regression), if done, indicating which were pre-specified.	N/A
**RESULTS**			
Study selection	17	Give numbers of studies screened, assessed for eligibility, and included in the review, with reasons for exclusions at each stage, ideally with a flow diagram.	Figure 1
Study characteristics	18	For each study, present characteristics for which data were extracted (e.g., study size, PICOS, follow-up period) and provide the citations.	Supplementary Table 1
Risk of bias within studies	19	Present data on risk of bias of each study and, if available, any outcome level assessment (see item 12).	N/A
Results of individual studies	20	For all outcomes considered (benefits or harms), present, for each study: (a) simple summary data for each intervention group (b) effect estimates and confidence intervals, ideally with a forest plot.	5-8, Tables, Supplementary Table
Synthesis of results	21	Present results of each meta-analysis done, including confidence intervals and measures of consistency.	5-8, Tables, Supplementary Table

Risk of bias across studies	22	Present results of any assessment of risk of bias across studies (see Item 15).	Supplementary Tables
Additional analysis	23	Give results of additional analyses, if done (e.g., sensitivity or subgroup analyses, meta-regression [see Item 16]).	N/A
**DISCUSSION**			
Summary of evidence	24	Summarize the main findings including the strength of evidence for each main outcome; consider their relevance to key groups (e.g., healthcare providers, users, and policy makers).	8-10
Limitations	25	Discuss limitations at study and outcome level (e.g., risk of bias), and at review-level (e.g., incomplete retrieval of identified research, reporting bias).	9-10
Conclusions	26	Provide a general interpretation of the results in the context of other evidence, and implications for future research.	10
**FUNDING**			
Funding	27	Describe sources of funding for the systematic review and other support (e.g., supply of data); role of funders for the systematic review.	10

From: Moher D, Liberati A, Tetzlaff J, Altman DG, The PRISMA Group (2009). Preferred Reporting Items for Systematic Reviews and Meta-Analyses: The PRISMA Statement. PLoS Med 6(6): e1000097. doi:10.1371/journal.pmed1000097

Supplementary Table S2. List of case-reported characteristics including presenting symptoms as well as treatment outcomes and comorbidities

Author(year)	Age	Patient's country	Gynecological history	Presenting symptom(period)	Combined abnormality	Number of doctor(s) for diagnosis	Treatment	Amount of drained fluid after treatment(mL)	Outcome
**Bekaert et al. (2018) [1]**	14 years old	Belgium	-	Chronic back pain<br>Abdominal pain	-	-	Hymenotomy	-	Improved
**Reggiani et al.(2018) [2]**	Neonate<br>(8 days old)	Mozambique	-	Fever, septic condition<br>Abdominal distention<br>Thoraco-abdominal respiration<br>Tense bulgingmembrane at vaginal introits,	Obstructive uropathy subsequent urosepsis, bladder perforation.	-	Laparotomy	-	Improved
**Elshiani et al. (2018) [3]**	27 years old	Kosovo	-	Problems with sexual intercourse.<br>Problems with pregnancy and cesarean delivery	-	-	Hymenectomy	-	Improved
**Ghafri et al. (2018) [4]**	15 years old	Oman	-	Amenorrhea<br>Abdominal distension	Obstructed hemivagina and ipsilateral renal anomaly (OHVIRA) syndrome	-	-<br>(Only recommended vaginoscopy and laparoscopy with vaginal septal resection)	-	-
**Brito et al. (2018) [5]**	19 years old	Brazil	-	Amenorrhea<br>Cyclic periodic lower abdominal and pelvic pain<br>Abdominal distension	-	-	Hymenotomy<br>Oral antibiotics	3000	Improved
**Kotter et al.(2017) [6]**	14 years old	USA	-	Intermittent lower abdominal pain(3weeks)<br>abdominal distension,<br>constipation,<br>breast tenderness	-	-	Hymenectomy	900	Improved
**Saleh et al. (2017) [7]**	12 years old	USA	Precocious puberty	Abdominal pain (2 weeks)<br>Urinary frequency (few weeks)<br>Non bloody emesis<br>Primary amenorrhea	Endometriosis	-	Hymenectomy	-	Improved
**Ramareddy et al.(2017) [8]**	Neonate (1 week)	India	Peter plus syndrome	Bilateral corneal opacity syndrome<br>Renal failure	Bilateral hydroureterone phrosis	-	Hymenectomy	-	Improved

**Ramareddy et al.(2017) [8]**	12 years	India	-	Abdominal pain<br>Bladder distension<br>Anuria	-	-	Hymenectomy	-	Improved
**Ramareddy et al.(2017) [8]**	9 years	India	-	Irritative and obstructive bladder	Vesicoureteral reflux	-	Stage repair	-	Improved
**Ramareddy et al.(2017) [8]**	Neonate<br>(2 weeks)	India	-	Pubic diastasis	Bladder exstrophy-epispadias complex	-	Hymenectomy	-	Improved
**Ramareddy et al.(2017) [8]**	14 years	India	-	Primary amenorrhea<br>Cyclic abdominal pain	-	-	Hymenectomy	-	Improved
**Ramareddy et al.(2017) [8]**	11 years	India	-	Primary amenorrhea<br>Cyclic abdominal pain	-	-	Hymenectomy	-	Improved
**Ramareddy et al.(2017) [8]**	12 years	India	-	Acute urinary retension	-	-	Hymenectomy	-	Improved
**Ramareddy et al.(2017) [8]**	11 years	India	-	Abdominal pain<br>Acute urinary retension	-	-	Abdominoperineal pull-through	-	Improved
**Zhang et al.(2017)[9]**	13 years old	China	-	Amenorrhea<br>Intra-labilal and pelvic mass(4months)	-	-	Hymenectomy	-	-
**Adam et al.(2017)[10]**	3 years old	South Africa	-	Recurrent UTI<br>Distended bladder	McKusick-Kaufman syndrome (MKKS)	-	Hymenectomy	-	-
**Elgyoum et al. (2016) [11]**	16 years old	Sudan	-	Cyclic lower abdominal pain, palpable suprapubic mass, primary amenorrhea	-	-	-	-	-
**Ramphul et al.(2016)[12]**	Neonate	UK	-	-	-	-	Hymenectomy	-	-
**Laghzaoui et al.(2016)[13]**	16 years old	Morocco	-	Cyclic pelvic pain(4years)<br>Primary amenorrhea	-	-	Hymenectomy and vaginoplasty	2000	Improved
**Nagaraj et al.(2016)[14]**	3 days old	India	-	Abdominal distension<br>Lower limb swelling	Bilateral hydronephrosis	-	Hymenectomy	-	Improved
**Tilahun et al.(2016)[15]**	23 days old	Ethiopia	-	Abdominal swelling<br>Urinary retention<br>Tachypnea (20 days)<br>Renal failure	Bilateral hydronephrosis,		Hymenectomy	1250	Died(cause: sepsis and AKI)
**Löllgen et al.(2016)[16]**	13 years old	Switzerland	-	Primary amenorrhea<br>Severe back pain<br>Urinary incontinence	-	-	Hymenectomy	1400	Improved
**Ossman et al.(2016)[17]**	16 years old	Egypt	3 hymenectomy	lower abdominal pain,<br>Urinary retention,	-	-	Hymenectomy	600	Improved

				Secondary amenorrhea (1.5 month)					
**Segal et al. (2015) [18]**	13 years old	USA	Labial Adhesions (treated topical estrogen)	Mild dysmenorrhea Inability to insert a tampon.	-	-	Progressive cervical dilators	-	Improved
**Segal et al. (2015) [18]**	16 years old	USA	-	Dysmenorrhea Inability to insert a tampon.	-	-	Progressive cervical dilators	-	Improved
**Al-Hunaidi et al.(2015)[19]**	23 years old	Kuwait	Hymenotomy for imperforate hymen	Urinary retention (1day) Amenorrhea(6months)	-	1	Hymenectomy	1200	Improved
**Glavan et al. (2015)[20]**	16 months old	Croatia	-	Redness and edema of vaginal ostium	-	2	Observation	-	-
**Lynn Coppola(2016)[21]**	adolescent	Ghana	Irregular menstruation	Urinary retention Leg pain	-	1	Hymenectomy	500	Improved
**Koyama-Sato et al.(2015)[22]**	16 years old	Japan	-	Cyclinc lower abdominal pain Primary amenorrhea	Transverse vaginal septum	2	Hymenectomy Vaginal septum repair	-	Vaginal canal adhesion(6months)
**Greene et al.(2015)[23]**	14 years old	USA	-	Fecal frequency and urgency(1year) Urinary frequency(1year) Pelvic pain(1year)	-	2	Hymenectomy	-	Improved
**George et al.(2015)[24]**	14 years old	germany	-	Abdominal pain(1year) Polyuria(1month) Primary amenorrhea	-	1	Hymenectomy Prophylactic antibiotics	2400	Reclosure of hymen(2months)
**Ulku et al.(2015)[25]**	15 years old	Turkey		Cyclic lower abdominal pain(1year)	Escobar syndrome	1	Eliptical hymenotomy	1000	Improved
**Plaza-Benhumea et al.(2014)[26]**	12 years old	Mexico	-	-	Langer–Giedion syndrome	1	Hymenoplasty Vaginal dilator therapy	-	Improved
**Ho et al.(2014)[27]**	14 years old	Australia	-	Lower abdominal pain(1day) Heavy bleeding per vagina(1day)	-	1	Cruciate hymenotomy	-	Improved
**Dilbaz et al.(2014)[28]**	13 years old	Turkey	-	Worsening lower abdominal pain(4months)	Transverse vaginal Septum	1	Hymenectomy Laparotomy because of rupture of abscess	200	Improved
**Fischer et al.(2014)[29]**	12 years old	Canada	-	Dysuria(3days) Constipation(3days) Perineal pain and pruritus(3days)	-	1	Hymenectomy	-	-

**Salhan et al.(2013)[30]**	12 years old	UK	-	Acute urinary retention(1day)<br>Lower abdominal pain(1day)<br>Dysuria(1day)	-	1	Cruciate hymenotomy	-	Improved
**Vitale et al.(2013)[31]**	3 days old	Italy	-	Abdominal mass	-	1	Hymenotomy	100	Improved
**Christodoulidou et al.(2013)[32]**	13 years old	UK	-	Lower abdominal pain(1day)<br>Urinary retention(1day)	-	3	Cruciate hymenotomy	-	Improved
**Mwenda AS.(2013)[33]**	14 years old	Kenya	-	Lower abdominal pain(1week)<br>Tenesmus(1week)	-	1	X-shaped hymenotomy	600	Improved
**Domany et al.(2013)[34]**	13 years old	Israel	-	Back pain(6months)<br>Loss of appetite(6months)	-	2	Hymenotomy	1000	Improved
**Rabani SM.(2013)[35]**	11 years old	Iran	-	Cyclie abdominal pain(6months)<br>Urinary retention	-	1	Hymenotomy	1000	Improved
**Rabani SM.(2013)[35]**	13 years old	Iran	-	Lower abdominal pain(6months)<br>Urinary retention	-	1	Hymenotomy	-	Improved
**Gupta et al.(2013)[36]**	11 years old	India	-	Lower abdominal pain (1day)<br>Dysuria(1day)	Transverse vaginal septum	1	Hymenectomy, Excision of transverse vaginal septum followed by dilatation	-	Fusion of vaginal septum(19days)
**Das et al.(2012)[37]**	15 years old	UK	-	Urinary retention<br>Cyclic lower abdominal pain(12moths)	-	2	Hymenectomy	400	Improved
**Mwampagatwa et al.(2012)[38]**	16 years old	Tanzania	-	Urinary retention(1day)<br>Lower abdominal pain	-	3	Hymenectomy	1500	Improved
**Sarathi et al.(2012)[39]**	18 months old	India	Central precocious puberty	Appearance of pubic hair and breast Enlargement(2months)	Vaginal atresia	1	Vaginal pull-through procedure,<br>GnRH agonist	-	Improved
**Eksioglu et al.(2012)[40]**	8 months old	Turkey	-	Fever of unknown origin	Bicornuate uterus	1	Cruciate hymenotomy	500	-
**Nagai et al.(2012)[41]**	35 days old	Japan	-	Abdominal distension<br>Bilateral leg edema<br>Venous distension<br>Renal failure	-	1	Hymenectomy	100	Improved
**Temizkan el al.(2012)[42]**	15 years old	Turkey	-	Lower abdominal pain(15days)	-	1	Central hymenotomy	700	Improved

				Back pain(15days)					
**Poll et al.(2011)[43]**	15 years old	Germany	-	Adominal pain(4days)	-	1	Hymenotomy	2000	-
**Kurdoglu et al.(2011)[44]**	13 years old	Turkey	-	Cyclic abdominal pain	-	1	Self limited(upper half of hymen)	-	Improved
**Ercan et al.(2011)[45]**	15 years old	Turkey	-	Lower abdominal pain<br>Urinary retention	-	2	Vertical hymenotomy	1000	Improved
**PDino et al.(2011)[46]**	12 years old	Croatia	-	Repeated urinary retention(1year)<br>Repeated abdominal pain(1year)<br>Renal failure	-	1	Hymenectomy	1500	Improved
**Khan et al.(2011)[47]**	19 years old	UK	Menstruation	Lower abdominal pain<br>Urinary retention<br>Dysuria(2weeks)<br>Urinary frequency(2weeks)	-	1	Cruciate hymenotomy	600	Improved
**Ameh et al.(2011)[48]**	2 days old	Nigeria	-	-	Persistent cloaca	-	Hymenotomy	-	Improved
**Ameh et al.(2011)[48]**	3 days old	Nigeria	-	-	Persistant cloaca	-	Hymenotomy	-	-
**Abu-Ghanem et al.(2011)[49]**	Adolescent	Israel	Simple vertical hymenotomy for imperforate hymen	Urinary retention(3days)<br>Dysuria(3days)<br>Lower abdominal pain(3days)	-	2	Hymenectomy	500	Improved
**Anselm et al.(2010)[50]**	14 years old	Nigeria	-	Urinary retention<br>Lower abdominal pain(2weeks)	-	2	Cruciate hymenotomy	1000	Improved
**Zhou et al.(2010)[51]**	Infant	China	-	-	Systemic aplasia cutis congenita	-	-	-	Died(cause:a sphyxia and dehydration)
**Adenuga et al.(2010)[52]**	Infant	USA	-	-	-	-	-	-	-
**Drakonaki et al.(2010)[53]**	12 years old	Greece	-	Abdominal discomfort(48hours)<br>Back pain(48hours)		1	Hymenotomy	-	Improved
**Kloss et al.(2010)[54]**	12 years old	USA	-	Lower abdominal pain(1month)		1	-	-	-
**Mou et al.(2009)[55]**	12 years old	Hong Kong	-	Cyclic lower abdominal pain(2months)	-	-	Hymenotomy	600	Improved
**Mou et al.(2009)[55]**	12 years old	Hong Kong	-	Lower abdominal pain(5months)	-	1	Hymenotomy	500	Improved

				Tenesmus(5months)<br>Dysuria(5months)					
**Mou et al.(2009)[55]**	13 years old	Hong Kong	-	Repeated lower abdominal pain(10months)	labial adhesion	-	Mini-hymenotomy	500	re-do hymenotomy (6th month after operation)
**Oakes et al.(2010)[56]**	14 years old	USA	-	Pelvic pain(5months)	Uterocervicovaginal septum	1	Hymenotomy, vaginal septoplasty	400	Improved
**Dhabalia et al.(2009)[57]**	25 years old	India	-	Primary infertility(4years)<br>Dyspareunia(4years)	Urethrovaginal fistula	1	Hymenotomy<br>Excision of fistula	-	Improved
**Partsinevelos et al.(2009)[58]**	12 years old	UK	-	Cystic tumor evaluation<br>Lower abdominal pain(2weeks)	-	2	Hymenectomy	-	Improved
**Basaran et al.(2009)[59]**	13 years old	Turkey	-	Lower abdominal pain<br>Urinary retention	-	1	Midline vertical hymenotomy	-	Improved
**Oguzkurt et al.(2009)[60]**	6 years old	Turkey	-	High fever<br>Abdominal pain	Urethrovaginal fistula<br>Primary vaginal calculus	2	Hymenotomy<br>Simple closure of the fistula<br>Extraction of vaginal canal	-	Improved
**Kalmantis et al.(2009)[61]**	15 years old	Greece	-	Lower abdominal pain(48hours)<br>Dysuria<br>Urinary frequency	-	1	Hymenectomy	1500	Improved
**Sharifiaghdas et al.(2009)[62]**	20 days old	Iran	-	Abdominal distension<br>Urinary retention	-	1	Small crossed hymenotomy	100	Improved
**Gyimadu et al.(2009)[63]**	12 years old	Turkey	-	Lower abdominal pain(24horus)<br>Urinary retention(24horus)	-	6	Cruciate hymenotomy	450	Improved
**Johal et al.(2009)[64]**	2 days old	UK	-	Large interlabial swelling<br>Urinary retention<br>Vomiting	-	1	Hymenotomy	100	Improved
**Lardenoije et al.(2009)[65]**	16 years old	Netherland	-	Primary amenorrhea<br>Cyclical abdominal pain(1year)	-	1	Hymenectomy	500	Improved
**Kumar et al.(2008)[66]**	11 years old	USA	-	Abdominal pain(2days)<br>Lower back pain(2days)<br>Dysuria	-	1	Hymenotomy	-	Improved

**Adali et al.(2009)[67]**	12 years old	Turkey	-	Urinary retention<br>Lower abdominal pain(1month)<br>Urinary hesistency(1week)	-	2	Cruciate hymenotomy	1000	Improved
**Hsu et al.(2008)[68]**	12 years old	Taiwan	-	Lower abdominal pain<br>Urinary retention	Hematocolpometra	3	Laparoscopic adhesiolysis<br>Cautery of the endometriosis	-	Improved
**Hijazeen R.(1998)[69]**	5 days old	Jordan	-	Diarrhea<br>Rapid breathing<br>Poor feeding	-	1	Hymenotomy	-	Improved
**Chang et al.(2007)[70]**	12 years old	Taiwan	-	Urinary retention(12hours)<br>Lower abdominal pain	-	2	Cruciate hymenotomy	-	Improved
**Buyukbayrak et al.(2008)[71]**	13 years old	Turkey		Urinary retention<br>Abdominal distention<br>Abdominal discomfort	-	2	T incision hymenotomy	300	Improved
**Deligeoroglou et al.(2007)[72]**	14 years old	Greece	-	Lower abdominal pain<br>Urine retention<br>Non-specific abdominal complaints	Transverse vaginal septum<br>Unicornuate uterus	1	Hymenectomy<br>Excision of transverse vaginal septum	200	Improved
**Dane et al.(2007)[73]**	16 years old	Turkey	-	Lower abdominal pain(7days)<br>Urinary retention(5days)	-	2	Cruciate hymenotomy	500	Improved
**Dane et al.(2007)[73]**	14 years old	Turkey	-	Urinary retention(2days)<br>Lower abdominal pain(2days)	-	1	Cruciate hymenotomy	-	Improved
**Khemchandani et al.(2007)[74]**	11 months old	India	-	Straining during urination<br>Repeated urinary retention	-	1	Cruciate hymenotomy	-	Improved
***Bajaj* et al.(2006)[75]**	Infant	UK	-	Bulging mass at the vaginal introitus	-	1	Cruciate hymenotomy	200–300	Improved
**Walsh et al.(2006)[76]**	14 years old	USA	-	Abdominal pain(2days)<br>Back pain(2days)<br>Urinary retention<br>Constipation	-	More than 7	Hymenectomy	1000	-
**Shen et al.(2006)[77]**	2 years old	Taiwan	-	Fever of unknown origin(4days)<br>Lethargy<br>Loss of appetite	-	2	Partial hymenotomy	150	Improved
**Levsky et al.(2006)[78]**	14 years old	USA	-	Repeated lower abdominal pain(2months)	Bicornuate uterus	3	Hymenotomy	500	Improved

**Aygun et al.(2006)[79]**	5 days old	Turkey	-	Abdominal distension<br>Renal failure	-	2	hymenotomy	150	Improved
**Sakalkale et al.(2005)[80]**	13 years old	New zealand	Familial history of imperforate hymen	Lower abdominal pain(36hours)<br>Urinary retention(36hours)	-	1	Hymenotomy	600	Improved
**Sakalkale et al.(2005)[80]**	14 years old	New zealand	Familial history of imperforate hymen	Cyclical lower abdominal Pain(3months)	-	1	hymenotomy	400	Improved
**Eskandar et al.(2004)[81]**	15 years old	UK	Familial history of imperforate hymen	Primary amenorrhoea<br>Cyclic lower abdominal pain	Duplex left kidney	2	Hymenectomy	-	Improved
**Eskandar et al.(2004)[81]**	17 years old	UK	-	Primary amenorrhoea<br>Cyclic lower abdominal pain	Angiomyolipoma arises primarily in the kidneys	-	Hymenectomy	-	Improved
**Wang et al.(2004)[82]**	-	Taiwan	-	chronic constipation and intermittent low back pain/6months	-	-	-	-	-
**Posner et al.(2005)[83]**	22 days old	USA	-	-	-	-	-	-	-
**Posner et al.(2005)[83]**	3months old	USA	-	-	-	-	-	-	-
**Posner et al.(2005)[83]**	4months old	USA	Familial history of imperforate hymen	-	-	-	-	-	-
**Posner et al.(2005)[83]**	7months old	USA	-	-	-	-	-	-	-
**Posner et al.(2005)[83]**	21months old	USA	-	-	-	-	-	-	-
**Posner et al.(2005)[83]**	2 years old	USA	-	-	-	-	-	-	-
**Posner et al.(2005)[83]**	2.1 years old	USA	Familial history of imperforate hymen	-	-	-	-	-	-
**Posner et al.(2005)[83]**	2.1 years old	USA	-	-	-	-	-	-	-
**Posner et al.(2005)[83]**	3.7 years old	USA	-	Dysuria	-	-	-	-	-
**Posner et al.(2005)[83]**	10.9 years old	USA	-	Vomiting<br>Lower abdominal pain	-	-	-	-	-
**Posner et al.(2005)[83]**	11.3 years old	USA	-	Abdominal pain<br>Dysuria	-	-	-	-	-
**Posner et al.(2005)[83]**	11.5 years old	USA	-	Abdominal pain(1month)<br>Back pain(1month)	-	-	-	-	-
**Posner et al.(2005)[83]**	11.7 years old	USA	-	Dysuria<br>Abdominal pain	-	-	-	-	-
**Posner et al.(2005)[83]**	12.2 years old	USA	-	Dysuria(1week)	-	-	-	-	-
**Posner et al.(2005)[83]**	12.2 years old	USA	-	Abdominal pain(2weeks)	-	-	-	-	-

**Posner et al.(2005)[83]**	12.3 years old	USA	-	Urinary retention(1day)	-	-	-	-	-
**Posner et al.(2005)[83]**	13.3 years old	USA	-	Lower abdominal pain(2months)	-	-	-	-	-
**Posner et al.(2005)[83]**	13.3 years old	USA	-	Urinary retention(2months)<br>Back pain(2months)<br>Abdominal pain(2months)	-	-	-	-	-
**Posner et al.(2005)[83]**	13.6 years old	USA	-	Cyclic abdominal pain(4months)<br>Dysuria(4months)	-	-	-	-	-
**Posner et al.(2005)[83]**	13.8 years old	USA	Familial history of imperforate hymen	Abdominal pain(3months)	-	-	-	-	-
**Posner et al.(2005)[83]**	13.9 years old	USA	-	Repeated abdominal pain(2weeks)<br>Dysuria(1day)	-	-	-	-	-
**Posner et al.(2005)[83]**	14.2 years old	USA	-	Abdominal pain(2days)	-	-	-	-	-
**Stone et al.(2004)[84]**	11 years old	USA	-	Abdominal pain(24horus)<br>Nausea(2days)<br>Urinary frequency(2days)<br>Dysuria(2days)	-	1	Hymenotomy	300	Improved
**Lim et al.(2003)[85]**	14 years old	Malaysia	Familial history of imperforate hymen	Cyclic abdominal pain(6months)	-	2	Hymenectomy	-	Improved
**Lim et al.(2003)[85]**	11 years old	Malaysia	Familial history of imperforate hymen	Urinary retention(1day)<br>Abdominal pain(1day)	-	1	Hymenectomy	800	Improved
**Chircop R.(2003)[86]**	13 years old	UAE	-	Lower abdominal pain(12hours)<br>Urinary retention(12hours)<br>Urinary frequency(2days)<br>Urinary urgency(2days)	-	1	hymenotomy	1000	Improved
**Wall et al.(2003)[87]**	12 years old	USA	-	Fever(3days)<br>Nausea(3days)<br>Vomiting(3days)<br>Lower abdominal pain(3days)	-	2	Hymenotomy	-	-
**Kumar et al.(2002)[88]**	-	India	-	Lower abdominal pain<br>Urinary retention	-	-	Hymenotomy	-	-
**Kumar et al.(2002)[88]**	-	India	-	Lower abdominal pain<br>Urinary retention	-	-	Hymenotomy	-	-

**Hu et al.(2001)[89]**	infant	USA	-	Non-immune hydrops Respiratory distress	-	2	Hymenotomy	-	Improved
**Botash et al.(2001)[90]**	9 years old	USA	Physical abuse	Child abuse referral and evaluation program evaluation	-	3	-	-	-
**Botash et al.(2001)[90]**	12 years old	USA	Physical abuse	Child abuse referral and evaluation program evaluation	-	1	-	-	-
**Botash et al.(2001)[90]**	3 years old	USA	Possible sex abuse	Child abuse referral and evaluation program evaluation(for sibling's abuse)	-	1	-	-	-
**Lok et al.(2001)[91]**	13 years old	China	-	Lower abdominal pain(2days) Urinary frequency constipation	-	1	Cruciate hymenotomy	-	Iatrogenic pyocolpos from needle puncture
**Stelling et al.(2000)[92]**	12 years old	USA	Familial history of imperforate hymen	Evaluation for familial history of imperforate hymen	-	1	Hymenectomy	-	Improved
**Stelling et al.(2000)[92]**	14 years old	USA	Familial history of imperforate hymen	Peritoneal signs	-	1	Hymenectomy	-	Improved
**Stelling et al.(2000)[92]**	14 years old	USA	Familial history of imperforate hymen	Evaluation for familial history of imperforate hymen	-	1	Hymenectomy	-	Improved
**Anguenot et al.(2000)[93]**	14 years old	Switzerland	-	Cyclic lower abdominal Pain(6months)	-	1	Radial hymenotomy	200	Improved
**Buick et al.(1999)[94]**	14 years old	UK	-	Back pain(72hours) Urinary incontinence(72horus) Constipation(72hours) Sudden chest pain with swelling of the upper torso	-	3	Cruciate hymenotomy	1000	Improved
**Ahmed et al.(1999)[95]**	12 years old	Australia	-	Repeated lower abdominal pain(3months)	Transverse vaginal septum	2	Cruciate hymenotomy Perforation and dilatation of septum	100	Improved
**Cicinelli et al.(1999)[96]**	-	Italy	Regular menstruation	Abdominal pain during menses(8months)	Didelphys uterus Ipsilateral renal agenesis	2	Hymenotomy Resectoscopic repair	-	Improved
**Hall DJ.(1999)[97]**	15 years old	UK	-	Lower abdominal pain(24hours)	-	1	Hymenotomy	1500	Improved

				Urinary retention					
**Bakos et al.(1999)[98]**	13 years old	Sweden	-	Lower abdominal pain(12hours)<br>Nausea(12hours)	-	2	X-shaped hymenotomy<br>Laparotomy for resection of ruptured right fallopian tube<br>Salpingostomy for obliterated left fallopian tube	400	Improved
**Pierce JT.(1999)[99]**	14 years old	USA	Sexual abuse	Urinary incontinence	-	1	Perforation and dilatation of hymen	-	-
**Kushnir et al.(1997)[100]**	13 years old	USA	-	Cyclic lower abdominal pain(3months)	-	1	Partial hymenectomy	400	Improved
**Tuncer et al.(1997)[101]**	8 months old	Turkey	-	Acute abdomen<br>Vomiting(4days)<br>Urinary retention(4days)<br>Constipation(4days)	Rupture of bladder	1	Puncture of the hymen<br>Laparotomy for persisting symptom<br>Bladder suturing	350	-
**Brevetti et al.(1997)[102]**	2 months old	USA	-	Abdominal distension(2days)<br>Irritability(2days)	-	2	Hymenotomy	240	Improved
**Bamshad et al.(1996)[103]**	5 years old	USA	Familial history of ulnar mammary syndrome	-	Ulnar Mammary Syndrome	-	-	-	-
**Bamshad et al.(1996)[103]**	11 years old	USA	Familial history of ulnar mammary syndrome	-	Ulnar Mammary Syndrome	-	-	-	-
**Bamshad et al.(1996)[103]**	6 years old	USA	Familial history of ulnar mammary syndrome	-	Ulnar Mammary Syndrome	-	-	-	-
**Robberecht et al.(1996)[104]**	13 years old	Belgium	-	Abdominal pain(4days)<br>Constipation(8days)	-	1	Diamond-shaped hymenotomy	1000	Improved
**Peterson-Sweeney et al.(1996)[105]**	13 years old	USA	-	Dysuria<br>Urinary retention<br>Back pain(1week)	-	2	Hymenectomy	800	Improved
**Loscalzo et al.(1995)[106]**	13 years old	USA	-	Lower abdominal pain(1week)<br>Constipation(1week)	-	3	Hymenotomy	1200	Improved
**Sanders et al.(1994)[107]**	19 years old	USA	Misdiagnosed as mullerian agenesis treated	Primary amenorrhea<br>Abdominal distention	-	-	Hymenectomy	1200	Improved

			with vaginal dilator						
**Lodh et al.(1994)[108]**	40 years old	India	-	Primary amenorrhea<br>Abdominal mass<br>Lower abdominal pain<br>Urinary retention	-	1	Hymenectomy<br>Removal of calculi in pelvis<br>Irrigation of vaginal cavity with antibiotic solution	-	Improved
**Usta et al.(1993)[109]**	13 years old	Lebanon	Familial history of imperforate hymen	Cyclic lower abdominal discomfort	-	1	Cruciate hymenotomy	1500	Improved
**Usta et al.(1993)[109]**	16 years old	Lebanon	Familial history of imperforate hymen	Cyclic lower abdominal discomfort	-	1	Hymenotomy	1000	-
**Usta et al.(1993)[109]**	8 years old	Lebanon	Familial history of imperforate hymen	-	-	1	Observation	-	-
**Nisanian AC.(1993)[110]**	14 years old	USA	-	Urinary retention(12hours)<br>Cyclic lower abdominal pain(2years)	-	1	Diamond-shaped hymenotomy<br>Hymenectomy<br>Prophylactic antibiotics	2600	Improved
**Yu et al.(1993)[111]**	16 years old	Taiwan		Urinary retention		1	Hymenotomy	1000	Improved
**Yu et al.(1993)[111]**	12 years old	Taiwan	-	Urinary retention<br>Lower abdominal pain	-	1	Cruciate hymenotomy	500	Improved
**Carlson et al.(1992)[112]**	14 years old	USA	Regular menstruation	Lower abdominal pain(3days)<br>Urinary frequency	Didelphic uterus with double vagina	2	Hymenectomy	500	Improved
**Gangopadhyay et al.(1992)[113]**	1 day old	India	-	Inability to pass meconium<br>A balloon shaped mass in vulval area	Imperforate anus	1	Cruciate hymenotomy<br>Anal cut back operation	20	-
**Catić et al.(1988)[114]**	16 years old	Yugoslavia	-	Abdominal pain(15days)<br>Pelvic pain(15days)<br>Flatulence(15days)<br>Constipation(15days)	-	1	Hymenotomy	1000	-
**Catić et al.(1988)[114]**	15 years old	Yugoslavia	-	Abdominal pain(2months)<br>Back pain(2months)	-	1	surgical incision of the imperforate hymen	1000	-
**Berkowitz et al.(1987)[115]**	6 years old	USA	Sexual abuse	Evaluation for sexual abuse	-	1	-	-	-
**Dickson et al.(1985)[116]**	14 years old	USA	Familial history of imperforate hymen	Abdominal pain(2days)	-	3	Partial hymenectomy	400	Improved

**Dickson et al.(1985)[116]**	14 years old	USA	Familial history of imperforate hymen	Syncope<br>Diarrhea<br>Vomiting	-	1	Partial hymenectomy	200	Improved
**Shaw et al.(1983)[117]**	Neonate	UK	-	Abdominal distention<br>Repiratory distress	-	-	-	-	-
**Shaw et al.(1983)[117]**	Neonate	UK	-	Anorectal atresia	Anorectal atresia	-	-	-	-
**Shaw et al.(1983)[117]**	Neonate	UK	-	Multiple anomalies	Cloaca<br>Congenital heart defect	-	-	-	Died(cause: cardiorespiratory distress)
**Shaw et al.(1983)[117]**	Neonate	UK	-	Multiple anomalies	Congenital heart defect	-	-	-	Died(cause: respiratory distress)
**Shaw et al.(1983)[117]**	Neonate	UK	-	Multiple anomalies	Bicornuate uterus<br>Anorectal atresia<br>Congenital heart defect<br>Sacral agenesis	-	-	-	Died(cause: respiratory distress)
**Shaw et al.(1983)[117]**	13 years old	UK	-	Abdominal pain<br>Pelvic mass	Right cystic dysplastic kidney<br>Right absent ureter<br>Anorectal atresia<br>Absent left radius	-	-	-	-
**Shaw et al.(1983)[117]**	9 years old	UK	-	Enuresis	-	-	-	-	-
**Shaw et al.(1983)[117]**	13 years old	UK	-	Abdominal pain<br>Perineal bulge	-	-	-	-	-
**Shaw et al.(1983)[117]**	13 years old	UK	-	Abdominal pain<br>Perineal bulge	-	-	-	-	-
**Shaw et al.(1983)[117]**	13 years old	UK	-	Abdominal pain<br>Perineal bulge	-	-	-	-	-
**Shaw et al.(1983)[117]**	14 years old	UK	-	Abdominal pain<br>Perineal bulge	-	-	-	-	-
**Shaw et al.(1983)[117]**	15 years old	UK	-	Appendicitis	-	-	-	-	-
**Shaw et al.(1983)[117]**	13 years old	UK	-	Appendicitis	Bilateral duplex ureter	-	-	-	-
**Shaw et al.(1983)[117]**	14 years old	UK	Regular	Appendicitis	Double genital	-	-	-	-

			menstruation		system				
**Shaw et al.(1983)[117]**	11 years old	UK	Regular mentruation	Abdominal pain<br>Abdominal mass	Double genital system	-	-	-	-
**Iuchtman et al.(1980)[118]**	8 years old	Israel	-	Lower abdominal pain	Left hypoplastic kidney with ectopic ureter<br>High aortic bifurcation	2	Hymenotomy	250	Improved
**Amortegui et al.(1979)[119]**	13 years old	USA	Known imperforate hymen	Abdominal crapms(2weeks)	Vaginal occlusion by an imperforate hymen	1	Hymenectomy	200	Improved
**Nidecker et al.(1978)[120]**	infant	Canada		Lower abdominal mass	Mild subluxation of the hips	1	Hymenotomy	-	Improved
**Bejanga BI.(1978)[121]**	16 years old	Cameroon		General discomfort(3weeks)<br>Dysuria(1week)<br>Constipation(1week)<br>Abdominal pain		2	Simple hymenotomy	1700	Reclosure(14 days)
**Kahn et al.(1975)[122]**	1 month old	USA	-	-	-	1	Self-limited	-	-
**Kahn et al.(1975)[122]**	Neonate	USA	-	-	-	1	Self-limited	-	-
**Gazit et al.(1975)[123]**	6 months old	Israel	-	Inability to pass stools(3days)	-	1	Hymenectomy<br>Prophylactic antibiotics	250	Improved
**Hansen et al.(1975)[124]**	13 years old	Denmark	-	Abdominal pain	-	1	Hymenectomy	500	Vaginal adenosis(2years)
**Hansen et al.(1975)[124]**	20 years old	Denmark	-	Primary amenorrhea<br>Abdominal pain	-	1	Hymenectomy	1100	Vaginal adhesion (1month)
**Hansen et al.(1975)[124]**	14 years old	Denmark	-	Acute abdominal pain<br>Abdominal tumor		1	Hymenectomy	1200	Vaginal adenosis (5weeks)
**CECUTTI A.(1964)[125]**	15 years old	Canada	-	Abdominal distention(6weeks)<br>Abdominal discomfort(6weeks)	-	2	Cruciate hymenotomy<br>Prophylactic antibiotics	3000	-
**WHITESELL et al.(1963)[126]**	14 years old	USA	-	Acute urinary retention(18hours)<br>Cyclic lower abdominal pain(2months)	-	1	Hymenectomy	600	Improved

**WHITESELL et al.(1963)[126]**	15 years old	USA	-	Lower abdominal enlargement<br>Cyclinc lower abdominal pain(6months)<br>Dysuria(6months)	-	1	Hymenectomy	950	Improved
**WHITESELL et al.(1963)[126]**	12 years old	USA	-	Acute urinary retention<br>Lower abdominal pain	-	1	Hymenectomy	150	Improved
**WHITESELL et al.(1963)[126]**	14 years old	USA	-	Back pain(4months)<br>Lower abdominal pain(4months)	-	1	Hymenectomy	1000	Improved
**HARPER J.(1961)[127]**	13 years old	Canada	-	Abdominal mass<br>Urinary frequency(1month)<br>Lower abdominal pain(2weeks)	-	1	Cruciate hymenotomy<br>Prophylactic antibiotics	3300	Improved
**HYATT HW Sr.(1960)[128]**	11 years old	USA	-	Cyclic lower abdominal pain(3moths)	-	1	Hymenectomy	325-300	Reclosure of hymen(2W)
**HYATT HW Sr.(1960)[128]**	14 years old	USA	Hymenotomy for imperforate hymen	Lower abdominal pain<br>Nausea	-	1	Hymenectomy	100	Improved
**HYATT HW Sr.(1960)[128]**	12 years old	USA	-	Lower abdominal pain	-	2	Hymenectomy	470	Improved
**HYATT HW Sr.(1960)[128]**	12 years old	USA	-	Abdominal mass<br>Urinary frequency	-	2	Hymenectomy	1150	Improved
**ROSENTHAL et al.(1958)[129]**	13 years old	USA	-	Abdominal distention(2days)<br>Urinary urgency(2days)<br>Urinary frequency(2days)	-	1	Hymenectomy	700	Improved
**ROSENTHAL et al.(1958)[129]**	14 years old	USA	-	Lower abdominal pain(6weeks)	-	1	Hymenectomy	800	-
**ROSENTHAL et al.(1958)[129]**	15 years old	USA	-	Cyclic lower abdominal pain(9months)	-	1	Hymenectomy	-.	Improved
**ROSENTHAL et al.(1958)[129]**	11 years old	USA	-	Lower abdominal mass<br>Abdominal discomfort(1week)	-	1	Hymenectomy	600	Improved
**WARNER et al.(1955)[130]**	16 years old	USA	Hymenotomy for imperforate hymen	Abnormal menstruation(3years)<br>Urinary retention(3year)	-	1	Hymenectomy<br>Dilatiation of vaginal orifice	-	-
**WARNER et al.(1955)[130]**	16 years old	USA	-	Primary amenorrhea<br>Cyclical abdominal pain(4years)		1	Hymenectomy<br>Laparotomy	400	Improved
**WARNER**	13 years old	USA	-	-	-	1	Hymenotomy	473	-

**et al.(1955) [130]**									
**WARNER et al.(1955) [130]**	15 years old	USA	-	Primary amenorrhea<br>Urinary retention	-	4	Hymenotomy	1000	Reclosure(15 days)
**WARNER et al.(1955) [130]**	17 years old	USA	-	Lower abdominal pain<br>Abdominal mass<br>Amenorrhea	-	2	Cruciate hymenotomy<br>Antibiotics	1000	Improved
**DANIEL WW.(1950)[131]**	14 years old	USA	-	Cyclic lower abdominal pain(1year)<br>Headache	-	2	Cruciate hymenotomy	470	Improved
**CRIGLER CM.(1946)[132]**	19 years old	USA	Menstruation	Scanty of menstruation<br>Dysmenorrhea<br>Dyspareunia	-	1	Surgical correction	-	Destruction of the urethral sphincter and bladder(2years)
**Doyle JC.(1942)[133]**	10 years old	USA	-	Dysuria(7months)<br>Vaginal fullness	-	-	Hymenectomy	-	Improved
**Doyle JC.(1942)[133]**	16 years old	USA	-	Primary amenorrhea	-	-	Hymenectomy	-	Improved
**Doyle JC.(1942)[133]**	14 years old	USA	Previously hymen opened	Amenorrhea	-	-	Hymenotomy	3000	Improved
**Doyle JC.(1942)[133]**	15 years old	USA	-	Lower abdominal cramps(6months)<br>Back pain(2weeks)<br>Constipation<br>Hot flushes	-	-	hymenectomy	500	-
**Doyle JC.(1942)[133]**	12 years old	USA	-	Abdominal pain<br>Legs drown up<br>Amenorrhea	-	-	Hymenectomy	600-1000	-
**Doyle JC.(1942)[133]**	12 years old	USA	-	Abdomina pain(2months)<br>Back pain(2months)<br>Legs pain(2months)<br>Constipation(1week)<br>Dysuria(3days)<br>Oliguria(3days)	-	-	Hymenotomy	1000	-
**Doyle JC.(1942)[133]**	17 years old	USA	-	Lower abdominal pain(1month)	-	-	Hymenectomy	1000	Improved
**Doyle JC.(1942)[133]**	15 years old	USA	-	Abdominal pain<br>Back pain<br>Abdominal mass<br>Dysuria(3weeks)<br>Frontal headache<br>Hot and cold flushes	-	-	Hymenectomy	800	Amenorrhea (1year)

**Doyle JC.(1942)[133]**	17 years old	USA	-	Abdominal pain(2weeks)<br>Abdominal distention(2weeks)	-	-	Hymenectomy	750	Developed cicatricial stenosis in upper vagina(8years)
**Doyle JC.(1942)[133]**	14 years old	USA	-	Lower abdominal pain(5days)<br>Rapid weight gain(10lb/4months)	-	-	Hymenotomy	700	Improved
**Doyle JC.(1942)[133]**	13 years old	USA	-	Abdominal pain (6weeks)<br>Abdominal distention (6weeks)<br>Back pain	-	-	hymenectomy	800	Improved
**Doyle JC.(1942)[133]**	13years old	USA	-	Suprapubic pain<br>Frequency, dysuria<br>Nocturia	-	-	Trochar puncture suction	-	-
**Doyle JC.(1942)[133]**	17 years old	USA	Regular menses from urethra	Incontinence of urine	Urethro-vaginal fistula	-	Hymenectomy<br>Reparing urethrovaginal fistula	-	Improved
**Doyle JC.(1942)[133]**	2 years old	USA	Hymenotomy 2 time previously for imperforate hymen	Vaginal irritation and tenderness			Hymenectomy	-	Improved
**Doyle JC.(1942)[133]**	12 years old	USA	-	Abdominal mass(6weeks)<br>Urinary retention	-	-	Hymenotomy	500	Improved
**Doyle JC.(1942)[133]**	17 years old	USA		Pelvic pressure<br>Amenorrhea	-	-	Hymenectomy	-	-
**Doyle JC.(1942)[133]**	15 years old	USA	-	Lower abdominal pain<br>Nausea<br>headache<br>Back pain<br>Listless	-	-	Hymenotomy	-	Improved
**Doyle JC.(1942)[133]**	16 years old	USA	-	-	-	-	Hymenectomy	-	Improved
**Doyle JC.(1942)[133]**	14 years old	USA	-	Epileptic attacks(2months)	-	-	Hymenotomy	-	Improved
**Doyle JC.(1942)[133]**	15 years old	USA	-	Crampy pain(18months)<br>Back pain<br>Swelling in introitus	-	-	Hymenotomy	-	Improved
**Bingham et al.(1938)[134]**	15 years old	USA	-	Amenorrhea<br>Abdominal mass(10months)	-	2	Longitudinal hymenoctomy	1500	Improved
**McIlroy et**	20 years old	UK	Familial history	Amenorrhea	-	1	Hymenectomy	-	Improved

**al.(1930)[135]**			of imperforate hymen	Abdominal mass<br>Cyclic abdominal pain<br>Frequent urination					
**McIlroy et al.(1930)[135]**	16 years old	UK	Familial history of imperforate hymen	Cyclic back and abdominal pain(6months)	-	1	Hymenectomy	-	Improved
**McIlroy et al.(1930)[135]**	14 years old	UK	Familial history of imperforate hymen	Familial history of imperforate hymen<br>Amenorrhea	-	1	Hymenectomy	945	Improved
**Roberts.(1873)[136]**	20 years old	UK	-	Abdominal mass<br>Dysuria	-	1	Hymenectomy	3000	Improved

Abbreviations: USA; United States of America, UK; United Kingdom, UTI; Urinary tract infection

Supplementary Table S3. List of perinatal case-reported characteristics including presenting symptoms as well as treatment outcomes and comorbidities

Authors (year)	Age(year)/parity	Gestational age at diagnosis (or impression)(week)	Presenting problem	Image modality(prenatal):(predictable) impression or image finding	Combined abnormality	Combined urinary problem(if it was not a presented problem)	Treatment	Amount of drained fluid after treatment(mL)	Outcome
**Rodriguez et al.(2017) [137]**	31/G2P1	37+3	Fetal abdominal cystic mass	US: fetal abdominal cystic mass with a fluid-debris level posterior to the bladder<br>Postnatal US: Hydrometrocolpos secondary to imperforate hymen<br>MRI: hydrometrocolpos		Left pelvic dilatation<br>Left ureteral distension<br>Poor kidney echogenocity	Hymenectomy	-	Improved
**Alici Davutogluet al.(2017)[138]**	35/G2P1	32	Fetal ambiguous genitalia	US : hydrocolpos<br>MRI : congenital imperforate hymen with hydrocolpos	-		Hymenotomy	200	Improved
**Nakajima et al.(2015)[139]**	36/G6P4	28	Fetal Pelvic mass	US : liquid pelvic mass<br>fMRI : imperforate hymen	-	-	Hymenotomy	-	Improved
**Ayaz et al.(2011)[140]**	19/G1P0	25	Fetal Abdominal cystic mass	US : hypoechoic mass	-	Left pevicailectasis	Hymenotomy	-	Improved
**Dosedla et al.(2011)[141]**	39/G5P3	37+1	Cardiotocographic exam. abnormality<br>Growth retardation	US : fetal hydrometrocolpos	Down syndrome	-	Self limited	-	-
**Bhargava et al.(2009)[142]**	32/G1P0	34	Fetal pelvic mass	US : pelvic mass	-	-	Hymenotomy	-	Improved
**Tseng et al.(2008)[143]**	32/G1P0	36	Fetal pelvic mass	US : pelvic mass<br>fMRI : congenital imperforate hymen with isolated hydrocolpos	-	Left hydronephrosis	Hymenotomy	80	Improved
**Yildirim et al.(2008)[144]**	28/G1P0	39	Fetal abdominal cystic mass	US : hydrometrocolpos and imperforate hymen	-	Mild dilated renal pevis	Hymenotomy	-	Improved
**Adaletli et al.(2007)[145]**	35/G2P1	18	Fetal bilateral hydronephrosis	US : megacystis-microcolon-intestinal hypoperistalsis Syndrome	-	-	Hymenotomy	200	Died(cause:urosepsis)

				fMRI : imperforated hymen with hydrocolpos					
**Ogunyemi D.(2001)[146]**	27/G4P2	22	Fetal uretocele	US : left ureterocele causing left hydronephrosis, dilation of the left ureter, and bladder outlet obstruction	-	Left hydronephrosis Calyceal system dilataion	Cystoscopy with endoscopic incision of the ureterocele and hymenotomy	-	Assymetric renal function Residual functional dilatation
**Winderl et al.(1995)[147]**	25/G1P0	25	Fetal renal abnomaly(echogenic right kidney, multiple pelvic cyst)	US : congenital imperforate hymen with hydrocolpos and a multiple dysplastic right kidney	Right multiple dysplastic kidney	-	Observation	-	-
**Ozturk et al.(2010)[148]**	26/-	40	-	US : bilateral hydronephrosis	Polydactyly of both feet and hands Laryngocele	-	Hymenectomy	100	Improved
**Karteris et al.(2010)[149]**	32/G3P1	29	Fetal abdominal cystic mass	US : pelvic mass, bilateral hydronephrosis	-	Mild bilateral hydronephrosis Ureteronephrosis	Hymenectomy	200	-
**El-Messidi et al.(2006)[150]**	35/G1P0	30	Oligohydramnios	US : bilateral hydronephrosis compressed renal tissue pelvic mass	McKusick-Kaufman syndrome	Bilateral hydronephrosis Large cystic abdominal mass	Hymenectomy	100	Improved
**Posner et al.(2005)[151]**	-	28	-	US : pelvic mass	-	-	-	-	-
**Krishna et al.(1998)[152]**	-	-	-	US : pelvic mass	-	-	Hymenectomy	200	-
**Jacquemyn et al.(1998)[153]**	26/G1P0	29	Fetal disproportionate fundal height Suspicion of polyhydramnios	US : fetal ascites distended uterus&vagina	-	Single umbilical artery Atrial septal defect type 2	Hymenotomy Transabdominal ascites aspiration	-	-

Abbreviations: US; Ultrasonography, G; Gravida, P; Para

Supplementary Table S4. Summary profiles of case-series reports about IH (not describe detail information of patients)

Author(year)	Age (Median)	Patient's country	Total number	IH	Combined abnormality	Treatment	Outcome
**Zhang et al. (2017) [154]**	-	China	-	3	Congenital vaginal atresia:	-	Improved
**Acar et al.(2007) [155]**	13.9±2.1 years	Turkey	65	65	Haematocolpos (100% of all patients)	Central oval incision and Foley catether inserted for balloon dilatation	Improved

Abbreviations: IH; Imperforate hymen

Supplementary Table S5. Combination of treatments for case-reported patients with imperforate hymen

Treatment	Total number of patients (n=236)
	Number of patients (%)
**Single kind of therapy**	**161(68.2%)**
Hymenotomy only	73(30.9%)
Hymenectomy only	74(31.4%)
Laparoscopic adhesiolysis only	2(0.8%)
Vaginal orifice dilatation only	2(0.8%)
Observation only	2(0.8%)
Laparotomy only	1(0.4%)
Vaginal septum repair only	1(0.4%)
GnRH agonist only	1(0.4%)
Perforation and dilatation of hymen only	1(0.4%)
Surgical incision of the imperforate hymen only	1(0.4%)
Unknown surgical correction only	1(0.4%)
Trochar puncture suction only	1(0.4%)
Abdominoperineal pull-through only	1(0.4%)
**Two kinds of therapy**	**19(8.1%)**
Hymenectomy + Vaginal septum repair	3(1.3%)
Hymenectomy + Laparotomy	2(0.8%)
Hymenectomy + Prophylactic antibiotics	2(0.8%)
Hymenectomy + Vaginoplasty	1(0.4%)
Hymenectomy + Repairing urethrovaginal fistula	1(0.4%)
Hymenectomy + Vaginal orifice dilatation	1(0.4%)
Hymenotomy + Prophyactic antibiotics	4(1.6%)
Hymenotomy + Anal cut back operation	1(0.4%)
Hymenotomy + Laparotomy	1(0.4%)
Hymenotomy + Simple closure of fistula + Extraction of vaginal canal	1(0.4%)
Hymenotomy + Excision of fistula	1(0.4%)
Hymenotomy + Vaginal septum repair	1(0.4%)
**Three kinds of therapy**	**3(1.3%)**
Hymenectomy + Hymenotomy + Prophylactic antibiotics	1(0.4%)
Hymenectomy + Removal of calculi in pelvis + Irrigation of vaginal cavity with antibiotic solution	1(0.4%)
Laparotomy + Puncture of the hymen + Bladder suturing	1(0.4%)

References

1. Bekaert T, Ramboer K. Hematometrocolpos Due to an Imperforate Hymen. J Belgian Soc Radiol 2018;102: 1–2.

2. Reggiani G, Pizzol D, Trevisanuto D, Antunes M. Successful management of giant hydrocolpos in a limited-resource setting. Oxford Medical Case Reports, 2018;7, 201–203.

3. Elshani B, Arifi H, Daci A. Microperforated Hymen Presenting Spontaneous Pregnancy with Cesarean Delivery and Hymenotomy Surgery: A Case Report. Maced J Med Sci. 2018: 6(3):528-530.

4. Ghafri AA, Fida A, Al-Gharras A. Obstructed Hemivagina and Ipsilateral Renal Anomaly (OHVIRA) Syndrome. Oman Med J 2018: 33(1); 69-71.

5. Brito LGO, Suto HS. Hematometrocolpos in a Teenager. NEJM 2018:378;8.

6. Kotter HC, Weingrow D, Canders CP. Hematometrocolpos in a Pubescent Girl with Abdominal Pain. Clin Prac Cases Emerg Med.2017:1(3):218-220.

7. Saleh R, Katzenbach G, Espinosa J. Hematometrocolpos Disguised as Abdominal Pain. J Emerg Med. 2017:53(5);e97-e99.

8. Ramareddy RS, Kumar A, Alladi A. Imperforate Hymen: Varied Presentation, New Associations, and Management. J Indian Assoc Pediatr Surg. 2017: 22(4); 207–210.

9. Zhang H, Qu H, Ning G, Cheng B, Jia F, Li X, et al. MRI in the evaluation of obstructive reproductive tract anomalies in paediatric patients. Clin Radiol 2017;72(7):612.e7-612.e15.

10. Adam A, Hellig J, Mahomed N, Lambie L. Recurrent Urinary Tract Infections in a Female Child With Polydactyly and a Pelvic Mass: Consider the McKusick-Kaufman Syndrome. Urology 2017;103:224–6.

11. Elgyoum AMA, Mohammed EAE, Mohammed AA. Case Report Diagnosing of Imperforate Hymen by Ultrasound. IJBMR. 2016:4(3); 27-30.

12. Ramphul M, Perry L, Bhatia C. Neonatal imperforate hymen with hydrocolpos. BMJ Case Rep. 2016 pii: bcr2016215434.

13. Laghzaoui O. Congenital imperforate hymen. BMJ Case Rep. 2016 pii: bcr2016215124

14. Nagaraj BR, Basavalingu D, Paramesh VM, Nagendra PD. Radiological Diagnosis of Neonatal Hydrometrocolpos- A Case Report. J Clin Diagn Res 2016;10(3):TD18-9.

15. Tilahun B, Woldegebriel F, Wolde Z, Tadele H. Hydrometrocolpos presenting as a huge abdominal swelling and obstructive uropathy in a 4 day old newborn: A diagnostic challenge. Ethiop J Health Sci 2016;26(1):89-91.

16. L öllgen RM, Sabo J, Mettler A, Liniger B, Berger S. Unique Presentation of Hematometrocolpos Mimicking Cauda Equina Syndrome: Severe Back Pain and Urinary Incontinence in an Adolescent Girl. J Emerg Med 2016;51(2):e19-23.

17. Ossman AME, El-Masry YI, El-Namoury MM, Sarsik SM. Spontaneous Reformation of Imperforate Hymen after Repeated Hymenectomy. J Pediatr Adolesc Gynecol 2016;29(5):e63-e65.

18. Segal TR, Fried WB, Krim EY, Parikh DP, Rosenfeld DL J Pediatr Adolesc Gynecol 2015;28:e21-22.

19. Al-Hunaidi O, El-Shazly M, Alkandari M, Ghobashy A, Alhajeri F, Allam A. Acute Urine Retention Caused by Hematocolpos After Failed Hymenotomy in 23 Year Old Female. Urol Case Rep 2015;3(2):50-1.

20. Glavan N, Haller H, Brnčić-Fischer A, Glavan-Gačanin L, Miletić D, Jonjić N. Imperforate hymen presenting as vaginal cyst in a 16-month-old child – considerations for an early diagnosis. Scott Med J

2016;61(1):48–50.

21. Coppola L. Unique Case of Imperforate Hymen. J Pediatr Adolesc Gynecol 2016;29(1):e1-3.

22. Koyama-Sato M, Hashida O, Nakamura T, Hirahara F, Sakakibara H. Case of early postoperative adhesion in a patient with molimina due to transverse vaginal septum concomitant with imperforate hymen. J Obstet Gynaecol Res 2015;41(7):1141–4.

23. Jacquemyn Y, Catte LD, Vaerenberg M. Fetal ascites associated with an imperforate hymen: sonographic observation. Ultrasound in Obstet Gynecol 1998;12(1):67–9.

24. Makris GM, Macchiella D, Vaidakis D, Chrelias C, Battista MJ, Siristatidis C. Abdominal Tumor in a 14-Year-Old Adolescent: Imperforate Hymen, Resulting in Hematocolpos—A Case Report and Review of the Literature. Case Reports in Obstet Gynecol 2015;2015:1–3.

25. Ulku MU, Yesim BT, Figen KS, Basar E, hizir K. Chronic abdominal pain in a patient with escobar syndrome. Gynecol Obstet Invest. 2015:79:69-72.

26. Plaza-Benhumea L, Valdes-Miranda JM, Toral-López J, Pérez-Cabrera A, Cuevas-Covarrubias S. Trichorhinophalangeal syndrome type II due to a novel 8q23.3-q24.12 deletion associated with imperforate hymen and vaginal stenosis Br J Dermatol 2014;171(6):1581–3.

27. Ho JW, Angstetra D, Loong R, Fleming T. Tuboovarian Abscess as Primary Presentation for Imperforate Hymen. Case Rep Obstet Gynecol 2014;2014:1–3.

28. Dilbaz B, Kiykac Altinbas S, Altinbas NK, Sengul O, Dilbaz S. Concomitant Imperforate Hymen and Transverse Vaginal Septum Complicated with Pyocolpos and Abdominovaginal Fistula. Case Rep Obstet Gynecol 2014;2014:1–4.

29. Fischer JW, Kwan CW. Emergency Point-of-Care Ultrasound Diagnosis of Hematocolpometra and Imperforate Hymen in the Pediatric Emergency Department. Pediatr Emerg Care 2014;30(2):128–30.

30. Salhan B, Omisore OT, Kumar P, Potter J. A Rare Presentation of Imperforate Hymen: A Case Report. Case Rep Urol 2013;2013:1–3.

31. Vitale V, Cigliano B, Vallone G. Imperforate hymen causing congenital hydrometrocolpos. J Ultrasound 2013;16(1):37–9.

32. Christodoulidou M, Kaba R, Oates J, Wemyss-Holden GD. Acute urinary retention in an adolescent girl and important learning points. BMJ Case Reports. 2013;2013. pii: bcr2013010361.

33. Mwenda AS. Imperforate Hymen - a care cause of acute abdominal pain and tenesmus: case report and review of the literature. Pan Afr Med J 2013;15:28.

34. Domany E, Gilad O, Shwarz M, Vulfsons S, Garty BZ. Imperforate hymen presenting as chronic low back pain. Pediatrics 2013;132(3):e768-70

35. Rabani SM. A Rare Non Urologic Cause for Urinary Retention; Report of 2 Cases. Nephrourol Mon 2013;5(2):766–8.

36. Gupta P, Gupta S, Jindal S, Chopra K, Sinha M, Arora A. Cervical dysgenesis with transverse vaginal septum with imperforate hymen in an 11 year old girl presenting with acute abdomen. JNMA J Nepal Med Assoc 2013;52(189):281-4.

37. Sayantana Das, Lisa Long. An unusual cause of acute urinary retention. British journal of hospital medicine. 2012;73(12):710-1.

38. Prabha Renu, Kumar Santosh, Kundu Saumyajyoti. Imperforate hymen presenting as acute urinary retention in an adolescent girl. International Journal of Reproduction, Contraception, Obstetrics and Gynecology.

2016:4484–5.

39. Vijaya Sarathi, Anand Naregal, Anurag Lila, Tushar Bandgar, Nalini Shah. Mucocolpos in a toddler: central precocious puberty with vaginal atresia. Endocrine Practice. 2012;18:e144-6.

40. Eksioglu Ayse Secil, Maden Hasim Ata, Cinar Gokce, Yildiz Yasemin Tasci. Imperforate Hymen Causing Bilateral Hydroureteronephrosis in an Infant with Bicornuate Uterus. Case Reports in Urology. 2012;2012:1–4.

41. Nagai Kozo, Murakami Yoshitaka, Nagatani Koji, et al. Life-threatening acute renal failure due to imperforate hymen in an infant. Pediatrics International. 2012;54(2):280–2.

42. Temizkan Osman, Kucur Suna Kabil, Agar Sema, Gozukara Ilay, Akyol Atif, Davas Inci. Virginity sparing surgery for imperforate hymen: report of two cases and review of literature. Journal of the Turkish German Gynecological Association. 2012;13(4):278–80. Doi: 10.5152/jtgga.2012.46.

43. Poll LW, Flake P. Alexander John L. Imperforate Hymen with Hematocolpometra. New England Journal of Medicine. 2011;365(2).

44. Kurdoglu Zehra, Kurdoglu Mertihan, Kucukaydin Zehra. Spontaneous Rupture of the Imperforate Hymen in an Adolescent Girl with Hematocolpometra. ISRN Obstetrics and Gynecology. 2011;2011:1–2.

45. Kouka S.c.n. Acute Urinary Retention by Hematocolpos in an Adolescent Girl with Imperforate Hymen: A Case Report. Journal of Nephrology and Urology Research. 2016;3(2).

46. Papeš Dino, Arslani Nuhi, Rajković Zoran, Altarac Silvio, Kopjar Miroslav. An Unusual Cause of Anuria and Hydronephrosis in a 12-Year-Old Girl. Renal Failure. 2011;33(5):540–3.

47. Khan Z. A., Rajesh U., Rastogi P., Joels L. A. Imperforate hymen: A rare case of secondary amenorrhoea. Journal of Obstetrics and Gynaecology. 2011;31(1):91–2.

48. Ameh Emmanuel A., Mshelbwala Philip M., Ameh Nkeiruka. Congenital Vaginal Obstruction in Neonates and Infants: Recognition and Management. Journal of Pediatric and Adolescent Gynecology. 2011;24(2):74–8.

49. Abu-Ghanem Sara, Novoa Rosa, Kaneti Jacob, Rosenberg Eran. Recurrent Urinary Retention Due to Imperforate Hymen After Hymenotomy Failure: A Rare Case Report and Review of the Literature. Urology. 2011;78(1):180–2.

50. Anselm Obio, Ezegwui Uzorh. Imperforate hymen presenting as acute urinary retention in a 14-year-old Nigerian girl. Journal of Surgical Technique and Case Report. 2010;2(2):84.

51. Tempark T, Shwayder TA. Aplasia cutis congenita with fetus papyraceus: report and review of the literature. Int J Dermatol 2012;51(12):1419–26.

52. Tanos V. Vaginal Septae and Imperforate Hymen. Female Genital Tract Congenital Malformations. 2014:231–9.

53. Drakonaki EE, Tritou I, Pitsoulis G, Psaras K, Sfakianaki E. Hematocolpometra Due to an Imperforate Hymen Presenting With Back Pain. J Ultrasound Med 2010;29(2):321-2.

54. Kloss BT, Nacca NE, Cantor RM. Hematocolpos secondary to imperforate hymen. Int J Emerg Med 2010;3(4):481–2.

55. Mou JW, Tang PM, Chan KW, Tam YH, Lee KH. Imperforate hymen: cause of lower abdominal pain in teenage girls. Singapore Med J 2009;50(11):e378-9.

56. Oakes MB, Hussain HK, Smith YR, Quint EH. Concomitant resorptive defects of the reproductive tract: a uterocervicovaginal septum and imperforate hymen. Fertil Steri 2010;93(1):268.e3-5.

57. Dhabalia JV, Nelivigi GG1, Satia MN2, Kakkattil S1, Kumar V1. Congenital Urethrovaginal Fistula With

Imperforate Hymen: A First Case Report. J Obstet Gynaecol Can 2009;31(7):652–3.

58. Partsinevelos GA., Rodolakis A, Loutradis D, Antsaklis A. Imperforate hymen is associated with elevated serum CA125 and CA19–9 levels: A reappraisal. J Obstet Gynaecol 2009;29(6):560–1.

59. Basaran M, Usal D, Aydemir C. Hymen Sparing Surgery for Imperforate Hymen: Case Reports and Review of Literature. J Pediatr Adolesc Gynecol 2009;22(4):e61-4.

60. Oguzkurt P, Ince E, Ezer SS, Temiz A, Demir S, Hicsonmez A. Primary vaginal calculus secondary to urethrovaginal fistula with imperforate hymen in a 6-year-old girl. J Pediatr Surg 2009;44(7):e11-3.

61. Fischer JW, Kwan CW. Emergency Point-of-Care Ultrasound Diagnosis of Hematocolpometra and Imperforate Hymen in the Pediatric Emergency Department. Pediatr Emerg Care 2014;30(2):128–30.

62. Schober J, Sharifiaghdas F, Abdi H, Pakmanesh H, Eslami N. Imperforate Hymen and Urinary Retention in a Newborn Girl. J Pediatr Adolesc Gynecol 2009;22(1):49–51.

63. Sharifiaghdas F, Abdi H, Pakmanesh H, Eslami N. Imperforate Hymen and Urinary Retention in a Newborn Girl. J Pediatr Adolesc Gynecol 2009;22(1):49–51.

64. Johal NS., Bogris S, Mushtaq I. Neonatal Imperforate Hymen Causing Obstruction of the Urinary Tract. Urology. 2009;73(4):750–1.

65. Lardenoije C, Aardenburg R, Mertens H. Imperforate hymen: a cause of abdominal pain in female adolescents. BMJ Case Reports. 2009;2009. pii: bcr08.2008.0722

66. Kumar K, Waseem M. An Uncommon Cause of Abdominal Pain in an Adolescent. South Med J 2008;101(10):1065–6.

67. Adali E, Kurdoglu M, Yildizhan R, Kolusari A. An overlooked cause of acute urinary retention in an adolescent girl: a case report. Arch Gynecol Obstet 2009;279(5):701-3.

68. Hsu KP, Chen CP, Chien SC, Hsu CY. Hematocolpometra Associated with an Imperforate Hymen and Acute Urinary Retention Mimicking a Pelvic Mass. Taiwan J Obstet Gynecol 2008;47(2):222–3.

69. Loscalzo IL, Catapano M, Loscalzo J, Sama A. Imperforate hymen with bilateral hydronephrosis: An unusual emergency department diagnosis. J Emerg Med 1995;13(3):337–9.

70. Joshi A, Tanwar H, Wagaskar V, Kaje Y, Satalkar MR. Imperforate Hymen with Acute Urinary Retention. J Case Reports 2015:382–6.

71. Buyukbayrak EE, Ozyapi AG, Karsidag YK, Pirimoglu ZM, Unal O, Turan C. Imperforate hymen: a new benign reason for highly elevated serum CA 19.9 and CA 125 levels. Arch Gynecol Obstet 2007;277(5):475–7.

72. Deligeoroglou E, Deliveliotou A, Makrakis E, Creatsas G. Concurrent imperforate hymen, transverse vaginal septum, and unicornuate uterus: a case report. J Pediatr Surg 2007;42(8):1446–8.

73. Dane C, Dane B, Erginbas M, Cetin A. Imperforate Hymen—A Rare Cause of Abdominal Pain: Two Cases and Review of the Literature. J Pediatr Adolesc Gynecol 2007;20(4):245–7.

74. Khemchandani S, Devra A, Gupta S. An unusual case of urinary tract obstruction due to imperforate hymen in an 11-month-old infant. Indian J Urol 2007;23(2):198.

75.Bajaj M, Becker M, Jakka SR. Imperforate hymen: a not so benign condition. Paediatr Child Health 2006;42(11):745-6

76. Walsh B, Shih R. An unusual case of urinary retention in a competitive gymnast. J Emerg Med 2006;31(3):279–81.

77. Shen MC, Yang LY. Imperforate Hymen Complicated with Pyocolpos and Lobar Nephronia. J Chin Med

Assoc 2006;69(5):224-7.

78. Levsky JM, Mondshine RT. Hematometrocolpos Due To Imperforate Hymen in a Patient with Bicornuate Uterus. AJR Am J Roentgenol. 2006;186(5):1469–70.

79. Aygun C, Özkaya O, Ayyýldýz S, Güngör O, Mutlu B, Küçüködük Ş. An unusual cause of acute renal failure in a newborn: hydrometrocolpos. Pediatr Nephrol 2006;21(4):572–3.

80. Usta IM., Awwad JT, Usta JA., Makarem MM, Karam KS. Imperforate hymen: Report of an unusual familial occurrence. Obstet Gynecol 1993;82(4):655–6.

81. Eskandar O, Wheble A. Imperforate hymen in two sisters associated with renal angiomyolipoma and duplex kidney. J Obstet Gynaecol 2004;24(8):922–3.

82. Anselm O, Ezegwui U. Imperforate hymen presenting as acute urinary retention in a 14-year-old Nigerian girl. Journal of Surgical Technique and Case Report 2010;2(2):84.

83. Posner JC, Spandorfer PR. Early Detection of Imperforate Hymen Prevents Morbidity From Delays in Diagnosis. Pediatrics 2005;115(4):1008–12.

84. Stone SM, Alexander JL. Images in clinical medicine. Imperforate hymen with hematocolpometra. N Engl J Med 2004;351(7):e6.

85. Usta IM, Awwad JT, Usta JA., Makarem MM, Karam KS. Imperforate hymen: Report of an unusual familial occurrence. Obstet Gynecol 1993;82(4):655–6.

86. Chircop R. A case of retention of urine and haematocolpometra. Eur J Emerg Med 2003;10(3):244–5.

87. Wall EM., Stone B, Klein BL. Imperforate hymen: a not-so-hidden diagnosis. Am J Emerg Med 2003;21(3):249–50.

88. Kumar K, Waseem M. An Uncommon Cause of Abdominal Pain in an Adolescent. South Med J 2008;101(10):1065–6.

89. Hu MX, Methratta S. An unusual case of neonatal peritoneal calcifications associated with hydrometrocolpos. Pediatr Radiol 2001;31(10):742–4.

90. Botash AS, Jean-LF. Imperforate Hymen: Congenital or Acquired From Sexual Abuse? Pediatrics 2001;108(3):E53.

91. Lok IH, Yip SK. Iatrogenic pyocolpos in a young girl with imperforate hymen. Aust N Z J Obstet Gynaecol 2001;41(1):104–5.

92. Stelling JR, Gray MR, Davis AJ, Cowan JM, Reindollar RH. Dominant transmission of imperforate hymen. Fertil Steril 2000;74(6):1241–4.

93. Anguenot JL, Ibecheole V, Salvat J, Campana A. Hematocolpos secondary to imperforate hymen, contribution of transrectal echography. Acta Obstet Gynecol Scand 2000;79(7):614–5.

94. Buick RG, Chowdhary SK. Backache: a rare diagnosis and unusual complication. Pediatr Surg Int 1999;15(8):586–7.

95. Ahmed S, Morris LL, Atkinson E. Distal mucocolpos and proximal hematocolpos secondary to concurrent imperforate hymen and transverse viginal septum. J Pediatr Surg 1999;34(10):1555–6.

96. Cicinelli E, Romano F, Didonna T, Schonauer LM, Galantino P, Di Naro E. Resectoscopic treatment of uterus didelphys with unilateral imperforate vagina complicated by hematocolpos and hematometra: case report. Fertil Steril 1999;72(3):553–5.

97. Hall DJ. An unusual case of urinary retention due to imperforate hymen. J Accid Emerg Med

1999;16(3):232–3.

98. Bakos O, Berglund L. Imperforate hymen and ruptured hematosalpinx: a case report with a review of the literature. J Adolesc Health 1999;24(3):226–8.

99. Pierce JT. A 14-year-old victim of sexual assault with an imperforate hymen and urethral meatus tear. J Emerg Nurs 1999;25(2):153–4.

100. Kushnir O, Garde K, Blankstein J. Rectal sonography for diagnosing hematocolpometra. A case report. J Reprod Med 1997;42(8):519-20

101. Tuncer R., Keskin E., Tunali N. Imperforate hymen as a cause of bladder perforation and intestinal obstruction. BJU International 1997;79(6):993–4.

102. Brevetti LS, Kimura K, Brevetti GR., Lawrence JP., Soper RT. Pyocolpos: Diagnosis and treatment. J Pediatr Surg 1997;32(1):110–1.

103. Bamshad M, Root S, Carey JC. Clinical analysis of a large kindred with the Pallister ulnar-mammary syndrome. Am J Med Genet 1996;65(4):325–31.

104. Robberecht E, Smets A, Winckel MV, Delens F. Arch Pediatr Adolesc Med 1996;150:993-4.

105. Peterson-Sweeney KL, Stevens J. 13-Year-Old Female With Imperforate Hymen. Nurse Pract 1996;21(8):90-4.

106. Loscalzo IL, Catapano M, Loscalzo J, Sama A. Imperforate hymen with bilateral hydronephrosis: An unusual emergency department diagnosis. J Emerg Med 1995;13(3):337-9.

107. Sanders RM, Nakajima ST. An unusual late presentation of imperforate hymen. Obstet Gynecol 1994;83:896-8.

108. Lodh U, Kumar S. Haematocolpos, Imperforate Hymen in an Adult Presenting with Primary Vaginal Calculi and Bladder Outlet Obstruction. Aust N Z J Obstet Gynaecol 1994;34(2):218-9.

109. Usta IM, Awwad JT, Usta JA, Makarem MM, Karam KS. Imperforate hymen: Report of an unusual familial occurrence. Obstet Gynecol 1993;82(4):655–6.

110. Nisanian AC. Hematocolpometra presenting as urinary retention. A case report. J Reprod Med 1993;38:57-60.

111. Yu TJ, Lin MC. Acute Urinary Retention in two Patients with Imperforate Hymen. Scand J Urol Nephrol 1993;27(4):543-4.

112. Carlson Robert L, Garmel Gus M. Didelphic uterus and unilaterally imperforate double vagina as an unusual presentation of right lower-quadrant abdominal pain. Ann Emerg Med 1992;21(8):1006–8.

113. Gangopadhyay AN, Pandit SK, Gopal SC. Anorectal anomaly (low) with imperforate hymen in a newborn. Indian Pediatr 1992;29:626-7.

114. Catić D, Lovrincević A, Cengić-Huković F, Klancević M. CT diagnosis of hematocolpometra. Comput Med Imaging Graph 1988;12(5):315-9.

115. Berkowitz CD, Elvik SL, Logan M. A Simulated "Acquired" Imperforate Hymen Following the Genital Trauma of Sexual Abuse. Clin Pediatr (Phila) 1987;26(6):307-9.

116. Dickson CA, Saad S, Tesar JD. Imperforate hymen with hematocolpos. Ann Emerg Med 1985;14:467-9.

117. Shaw LM, Jones WA, Brereton RJ. Imperforate hymen and vaginal atresia and their associated anomalies. J R Soc Med 1983;76:560-6.

118. Iuchtman M, Assa J, Blatnoi I, Ezagui L, Simon J. Urometrocolpos Associated With Retroiliac Ureter. J Urol 1980;124(2):283–5.

119. Amortegui AJ, Kanbour AI, Silverstein A. Diffuse vaginal adenosis associated with imperforate hymen. Obstet Gynecol 1979;53(6):760-2.

120. Nidecker AC, Humphry A. Peritoneal calcification in a neonate with imperforate hymen. J Can Assoc Radiol 1978;29:277-9.

121. Bejanga BI. Hematocolpos with imperforate hymen. Int Surg 1978;63:97-9.

122. Kahn R, Duncan B, Bowes W. Spontaneous opening of congenital imperforate hymen. J Pediatr 1975;87(5):768–70.

123. Gazit E, Frand M, Mashiah S, Rotem Y. Imperforate Hymen Causing Pyocolpos in an Infant. Clin Pediatr (Phila) 1975;14(4):414-5.

124. Hansen K, Egholm M. Diffuse Vaginal Adenosis. Three Cases Combined With Imperforate Hymen And Haematocolpos. Acta Obstet Gynecol Scand 1975;54(3):287-92.

125. Cecutti A. Hematocolpos with imperforate hymen. Can Med Assoc J 1964;90:1420-1.

126. Whitesell Jr, White WJ. The imperforate hymen with occult menstruation. Surg Gynecol Obstet 1963;117:618-22

127. Harper J. Hematocolpos with imperforate hymen. Am J Obstet Gynecol.1961;82(2):271–6.

128. Hyatt HW. Imperforate hymen: review of the literature and report of four additional cases. Am Pract Dig Treat 1960;11:1016-21.

129. Rosenthal AH., Block RE., Lapin A. The imperforate hymen as a cause of “abdominal tumor.” Am J Surg 1958;95(3):487–8.

130. Warner RE., Mann RM. Hematocolpos With Imperforate Hymen: report of five cases. Obstet Gynecol 1955;6(4):405-9.

131. Daniel WW. Imperforate hymen with hematocolpos; case report. South Surg 1950;16:473-5.

132. Crigler CM. Urological complications following operation for imperforate hymen. J Urol 1946;56(2):211–22.

133. Doyle JC. Imperforate Hymen: With and Without Hematocolpos: A Review of The Literature And A Report of Twenty Cases. Cal West Med 1942;56:242-7

134. Bingham EM, Blevins WJ. Hematocolpos Due to Imperforate Hymen. Cal West Med 1938;49:147..

135. McIlroy DL, Ward IV. Three Cases of Imperforate Hymen Occurring in One Family. Proc R Soc Med 1930;23(5):633-4.

136. Roberts DL. Short Notes of a Case of Imperforate Hymen: In which Upwards of One Hundred Ounces of Retained Menstrual Fluid were Evacuated. BMJ 1873;2(668):456–7.

137. Rodriguez RG, Gonzalez JP, Delgado RG, Guedes AR, Alvarado ML, Castellano MM, Hernandez JAG. Fetal hydrometrocolpos and congenital imperforate hymen: Prenatal and postnatal imaging features.J Clin Ultrasound. 2018;46:549-552.

138. Alici Davutoglu E, Yuksel MA, Yurtkal A, Temel Yuksel I, Adaletli IM, Madazli R. Prenatal diagnosis of isolated foetal hydrocolpos secondary to congenital imperforate hymen mimicking ambiguous genitalia. J Obstet Gynaecol 2017;37:248-9.

139. Nakajima E, Ishigouoka T, Yoshida T, Sato T, Miyamoto T, Shirai M, et al. Prenatal diagnosis of congenital imperforate hymen with hydrocolpos. J Obstet Gynaecol 2014;35(3):311–3.

140. Ayaz UY, Dilli A, Api A.. Ultrasonographic diagnosis of congenital hydrometrocolpos in prenatal and newborn period: a case report. Med Ultrason 2011;13(3):234-6.

141. Dosedla E, Kacerovsky M, Calda P. Prenatal diagnosis of hydrometrocolpos in a down syndrome fetus. J Clin Ultrasound. 2011;39(3):169–71.

142. Bhargava P, Dighe M. Prenatal US diagnosis of congenital imperforate hymen. Pediatr Radiol 2009;39(9):1014.

143. Tseng JJ, Ho JY, Chen WH, Chou MM. Prenatal Diagnosis of Isolated Fetal Hydrocolpos Secondary to Congenital Imperforate Hymen. J Chin Med Assoc. 2008;71(6):325–8.

144. Yildirim G, Gungorduk K, Aslan H, Sudolmus S, Ark C, Saygin S. Prenatal diagnosis of imperforate hymen with hydrometrocolpos. Arch Gynecol Obstet 2008;278(5):483-5.

145. Adaletli I, Ozer H, Kurugoglu S, Emir H, Madazli R. Congenital Imperforate Hymen with Hydrocolpos Diagnosed Using Prenatal MRI. AJR Am J Roentgenol 2007;189(1): W23-5.

146. Ogunyemi D. Prenatal Sonographic Diagnosis of Bladder Outlet Obstruction Caused by a Ureterocele Associated with Hydrocolpos and Imperforate Hymen. Am J Perinatol 2001;18(01):15–22.

147. Winderl LM, Silverman RK. Prenatal diagnosis of congenital imperforate hymen. Obstet Gynecol 1995;85(5):857–60.

148. Ozturk H, Yazici B, Kucuk A, Senses DA. Congenital imperforate hymen with bilateral hydronephrosis, polydactyly and laryngocele: A rare neonatal presentation. Fetal Pediatr Pathol 2010;29(2):89–94.

149. Karteris E, Foster H, Karamouti Maria, Goumenou Anastasia. Congenital Imperforate Hymen with Hydrocolpos and Hydronephrosis associated with Severe Hydramnios and Increase of Maternal Ovarian Steroidogenic Enzymes. J Pediatr Adolesc Gynecol 2010;23(3):136–41.

150. El-Messidi A, Fleming NA. Congenital Imperforate Hymen and Its Life-threatening Consequences in the Neonatal Period. Journal of Pediatric and Adolescent Gynecology. 2006;19(2):99–103.

151. Posner JC, Spandorfer PR. Early Detection of Imperforate Hymen Prevents Morbidity From Delays in Diagnosis. Pediatrics 2005;115(4):1008–12.

152. Krishna A, Arora NK. Imperforate hymen with hydrocolpos. Indian Pediatr. 1998;35(10):1028.

153. Jacquemyn Y, De Catte L, Vaerenberg M. Fetal ascites associated with an imperforate hymen: sonographic observation. Ultrasound Obstet Gynecol 1998;12:67-9.

154. Zhang M, Zhang M, Li G, Xu C. Congenital Vaginal Atresia: A Report of 39 Cases in a Regional Obstetrics and Gynecology Hospital. J Huazhong Univ Sci Technol [Med Sci] 2017;37:928-932.

155. A Acar, O Balci, R Karatayli, M Capar, MC Colakoglu. The treatment of 65 women with imperforate hymen by a central incision and application of Foley catheter. BJOG 2007;114:1376-1379.
